# ﻿Fungal fairy rings: history, ecology, dynamics and engineering functions

**DOI:** 10.3897/imafungus.16.138320

**Published:** 2025-02-17

**Authors:** Maurizio Zotti, Giuliano Bonanomi, Stefano Mazzoleni

**Affiliations:** 1 Department of Agricultural Sciences, University of Naples Federico II, Portici NA, via Università 100, Naples, Italy University of Naples Federico II Portici Italy; 2 Task Force on Microbiome Studies, University of Naples Federico II, Portici NA, via Università 100, Naples, Italy University of Naples Federico II Portici Italy

**Keywords:** *Basidiomycetes*, ecosystem engineer, fungal fronts, mycelial mats, self-DNA inhibition, vegetation patterns

## Abstract

Fungal fairy rings (FFR) are fascinating natural phenomena that have intrigued people and scientists for centuries. These patterns, often represented by circular distributions of altered vegetation, are found in grasslands and forest habitats. Fairy rings occur when fungi grow radially in the soil, raising from a central point, progressively degrading organic matter and thus affecting vegetation. The observation of such spatial patterns allows mycologists to conduct an in-depth analysis of the role of fungi in ecosystems.

This review presents the current knowledge and scientific advancement of the studies of FFRs. An historical appraisal from the most representative pioneer studies until recent works is presented in different scientific fields, including microbiology, chemistry, botany and ecology. Based on a deep analysis of bibliographic data, we synopsised different aspects of FFRs: i) history of studies, ii) taxonomy, iii) ecology (environmental conditions and biogeography), iv) classification of vegetation patterns, v) spatial dynamics, vi) role as ecosystem engineer (impact on soil chemistry, plants and microbiota).

In conclusion, beside still open research areas requiring further investigation, a schematic functional model of fungal fairy rings is proposed, in which on one hand the dynamics of the fungal mycelium is explained by self-DNA accumulation and the build-up of autotoxicity. On the other hand, the effects of fungi on plants are related to the intermingled and differently spatially distributed effects of hydrophobicity, phytotoxicity and phytostimulation.

## ﻿Introduction

The term fairy ring originates from a legacy of mysticism surrounding natural phenomena in ancient times. Despite its mythical connotations, the term has been preserved in scientific literature to describe two distinct phenomena: patterns of verdant or dead vegetation in grasslands and the circular arrangement of sporophores on forest floors. In this work, we adopt the term fungal fairy ring (FFR) as proposed by Getzin et al. (2021), while acknowledging that this pattern might be more accurately referred to as a fungal front.

**Figure 1.**
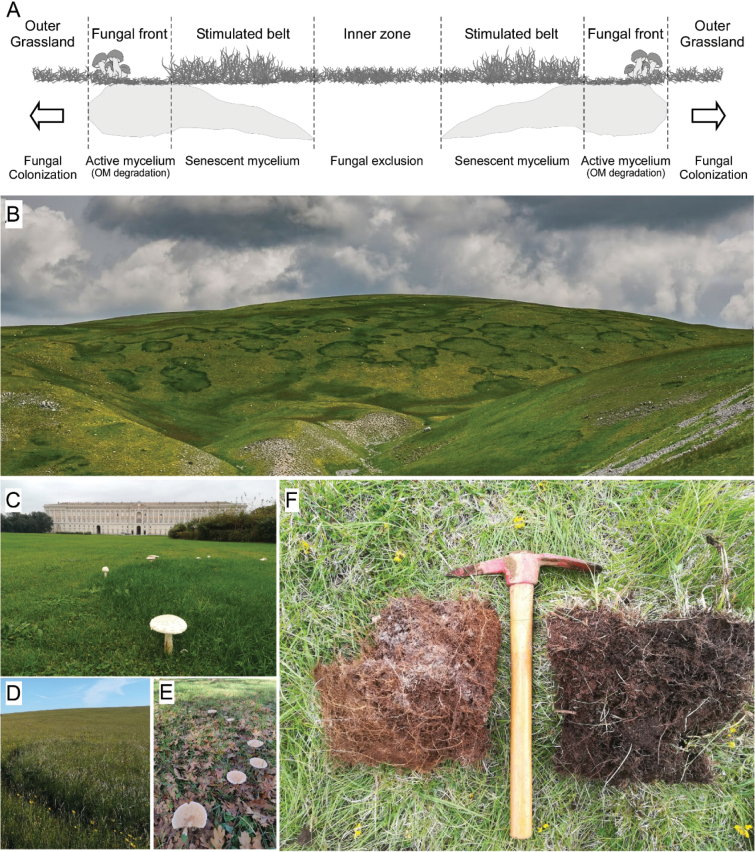
**A** Cross-section of a fungal fairy ring (FFR) transect providing a visual representation of the mycelial mat distribution in the soil, with arrows representing growth direction **B**FFRs of *Agaricuscrocodilinus* in Monte Pratello subalpine grassland, Rivisondoli, Abruzzo, Italian Apennines. (Photo by Franco Carnevale) **C**FFR of *Amanitavittadini* in managed grassland of Reggia di Caserta, Campania **D**FFR caused by *Agaricusarvensis* in species rich managed grassland of Monte Rogedano, Marche **E**FFR of *Infundibulicybegeotropa* in *Quercuscerris* woodland of Atina, Lazio, (Photo by Tiziana Pagnani) **F** soil of subalpine grassland affected by fungal front of *Calocybegambosa* FR in Monte Mutria, Campania Italian Apennines. On the left, soil densely occupied by mycelial mat, on the right unaffected soil.

FFRs are the result of radial expansion of fungal fronts within the soil. They are typically formed by basidiomycete fungi (Couch 1962; Halisky and Peterson 1970). Soil occupied by fungal mycelium is characterised by a dense mycelial mat, friable texture, whitish colouration and a distinctive fungal odour (Fig. 1A, F). On a broader scale, these dense mats of mycelium exhibit a toroidal structure that seasonally expands along its outer edges (Parker-Rhodes 1955).

At the boundaries of these expanding mats, the soil undergoes significant perturbation as mycelial growth alters its physicochemical properties. These changes, in turn, impact surrounding plants and microbial communities. In grasslands, FFR fungi are well-known for producing greener bands of grass cover (Fig. 1B), making them one of the most striking phenomena created by soil microbes at a landscape level. In woodland ecosystems, FFRs manifest as sporophores arranged in circular formations (Gregory 1982; Dowson et al. 1989; Peter 2006) (Fig. 1C–E). Occasionally, the extensive growth of fungal fronts obscures the ring-like pattern when viewed at close range, but it becomes distinctly visible from a landscape perspective (Fig. 1B).

The name “fairy rings” reflects the characteristic circular shape caused by fungal fronts’ interaction with vegetation. However, variations such as ribbons, arcs or rotors have been documented, arising from fragmentation and coalescing patterns during fungal front expansion (Shantz and Piemeisel 1917; Stevenson and Thompson 1976). Over the past decades, FFRs have inspired a wide array of studies. These investigations have explored their role in creating regular vegetation patterns (Parker-Rhodes 1955; Karst et al. 2016; Allegrezza et al. 2022), their pathogenic effects in managed grasslands (Lebeau and Hawn 1961; Fidanza 2007b; Caspar and Spiteller 2015; Fidanza et al. 2016) and their potential as genetic resources for human applications (Kawagishi 2018; Takano et al. 2019; Ito et al. 2020). Additionally, their impact on soil microbiota (Ohara and Hamada 1967; Marí et al. 2020; Zotti et al. 2020; Zotti et al. 2021) and utility as indicators for belowground fungal mycelium detection (Mallett and Harrison 1988; Dowson et al. 1989; Abesha et al. 2003; Hiltunen et al. 2019; Hiltunen et al. 2021) have been explored extensively.

This review aims to provide an updated synthesis of current understanding of FFRs and their associated complex phenomena in ecosystem functioning. Following a historical overview, we present a comprehensive analysis of studies on the mechanisms driving FFR formation and occurrence, their role as ecosystem engineers influencing vegetation composition and diversity and their effects on soil microbiota.

## ﻿History of FFRs

**Figure 2.**
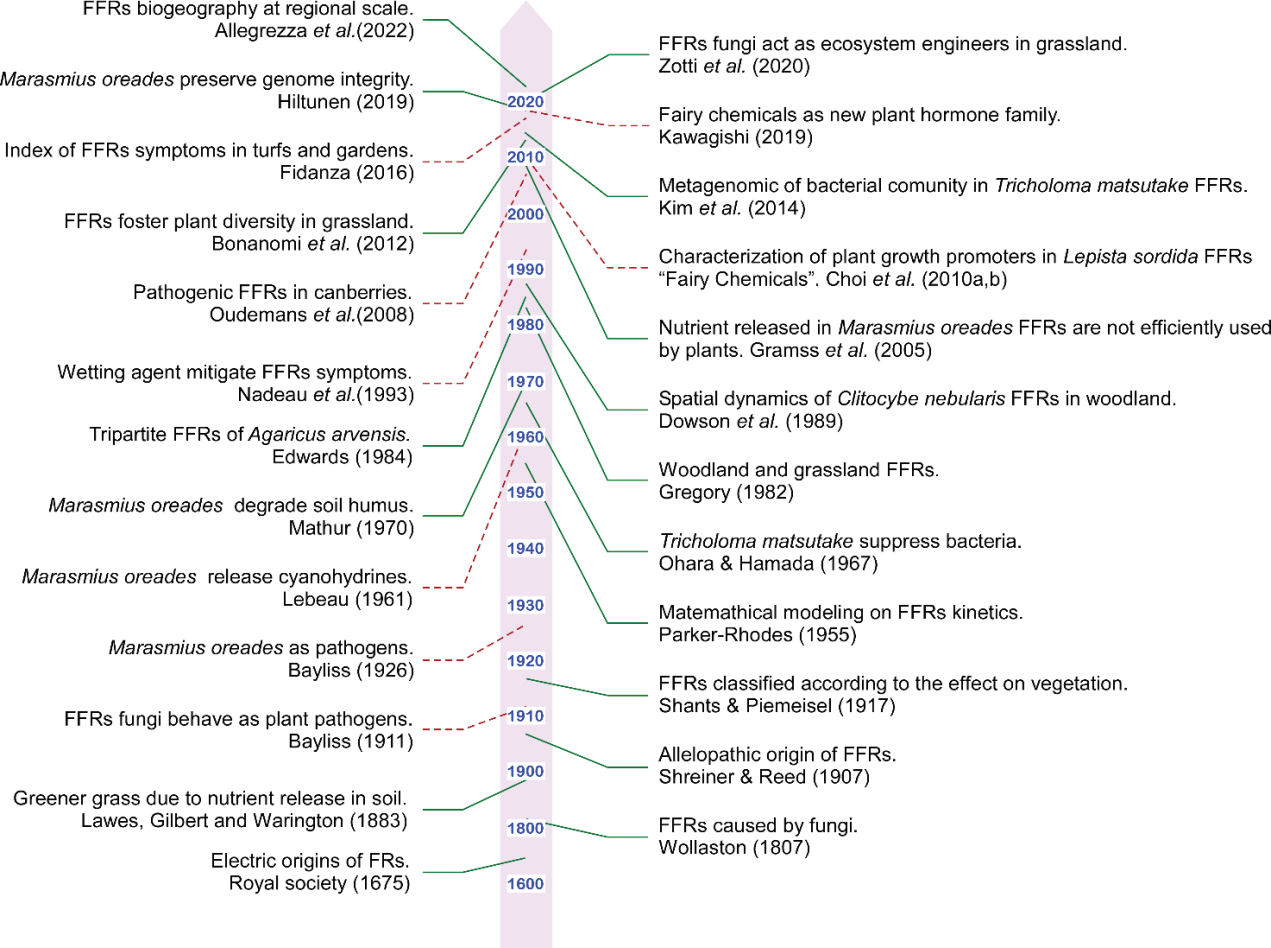
Timeline of most representative published works on FFRs, since 1600. Green solid lines refer to studies in fungal ecology and biology. Red dashed lines indicate research work in plant pathology and agricultural applications.

The history of fungal fairy rings (FFRs) begins with the striking regularity of their sporophore arrangements and vegetation changes, which gave rise to folk beliefs attributing the phenomenon to magical rituals. Terms such as “Cerchio delle streghe” in Italy, “Rond de sorcièr” in France and “Corro de brujas” in Spain reflect these mystical associations, as many believed the rings were the result of sorcerers’ activities. A portion of these beliefs, particularly from European traditions, has been reviewed in Shantz and Piemeisel (1917). In Fig. 2, we describe the most representative works aimed at explaining the phenomenon, both in the fields of ecology and biology and in those focused on plant pathology and production.

From a scientific perspective, early researchers proposed various potential causes for FFRs, including activities of subterranean mammals, ant colonies, deposits of faeces and urine by herbivores (Shantz and Piemeisel 1917). More than two centuries ago, however, scientists, such as Wollaston (1807) and Evershed (1884), began to identify fungi growing belowground as the primary cause of FFRs.

**Figure 3.**
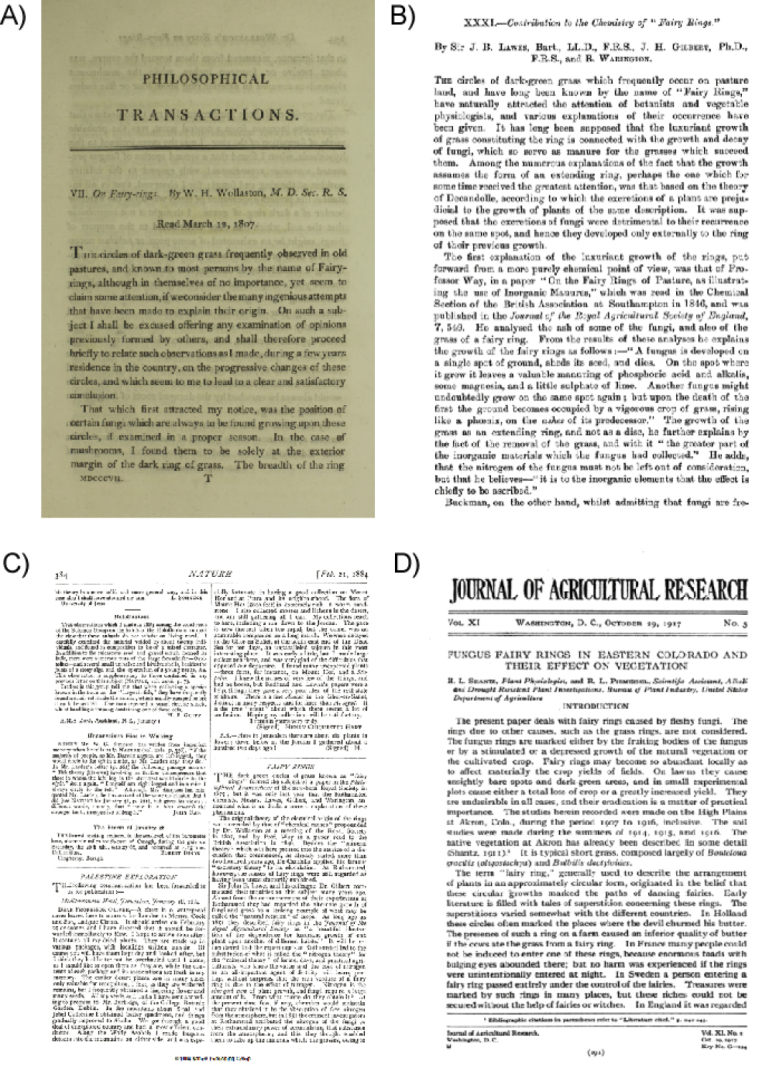
Cover pages of some representative historical publications on fungal fairy rings **A** the work of W. H. Wollaston in Philosophical Transactions of Royal Society, first to describe fungal origins of the rings in 1807 **B** contribution to the chemistry of fairy rings in the Journal of the Chemical Society in 1883 by J. B. Lawes, J. H. Gilbert and R. Warington **C** the work of H. Evershed published in Nature 1884 collecting evidence of nitrogen accumulation as biostimulant of vegetation in the ring pattern **D** the review published by H. L. Shantz and R. Piemeisel in 1917 in the Journal of Agricultural Research.

The processes by which FFR fungi expand and affect vegetation were debated for decades (Fig. 3). In 1675, scientists from the Royal Society hypothesised that the dark green circles associated with FFRs had “electrical origins” tied to lightning bolts (Evershed 1884). Later, Wollaston (1807) proposed that nitrogen enrichment in the soil was responsible for the characteristic verdant vegetation. Similarly, John Thomas Way of the Agricultural College of Cirencester (1846) attributed greener vegetation to the presence of potash and nitrogen in fungal mycelium. Definitive evidence for the chemical basis of FFR patterns came from Lawes et al. (1883), who demonstrated nitrogen accumulation in FFRs formed by *Calocybegambosa* (Fr.) Donk and *Marasmiusoreades* (Bolton) Fr. in the gardens of Rothamsted Experimental Station. Evershed (1884) further clarified FFR knowledge by documenting the ability of these rings to alter plant communities, with degraded vegetation inside the ring and flourishing growth on its outer edges.

Swiss botanist and mycologist De Candolle (1830–1832) contributed significantly to understanding FFRs by exploring their peculiar patterns. He investigated the deficiency of plant species and cover within FFRs, the confinement of fungi to the outer edge due to excreted residues from older mycelium and the outward expansion of rings driven by spore dispersal and poor germination in central areas. De Candolle hypothesized that fungal excretions impaired recolonisation at the centre, leading to the characteristic doughnut-like shape of FFRs. Despite their innovative nature, these theories were initially overlooked due to limited experimental evidence available at the time (Way 1847).

In 1910, Molliard advanced the understanding of FFRs through observations on the cliffs of Saint-Cast, France. He identified three distinct zones within FFRs bordered by *M.oreades* sporophores: a greener outer edge, a bare central zone with whitish soil and an inner ring of taller, greener vegetation. Molliard attributed these patterns to nutrient cycling, particularly ammonia enrichment from fungal senescence and water sequestration by fungal mycelium in the bare zone. His methods mirrored those of Lawes et al. (1883) and highlighted the occurrence of ammonia enrichment in the greener vegetation and higher ammonia levels in the bare zone.

Bayliss (1911) added further insights by suggesting a parasitic potential of *M.oreades* in nutrient-poor grasslands. He observed chemical exudates affecting grass roots and explained the greener belt of vegetation because of nitrogen enrichment from proteolytic enzymes. Additionally, Bayliss investigated the expansion rate of FFRs, conditions promoting their formation and the interaction of fungal fronts over three years. He offered explanations for the development of bare zones and the consistent ring-shaped pattern.

In 1917, Shantz and Piemeisel published a seminal work on FFRs formed by *Agaricuspraerimosus* Peck and *Calvatiacyathiformis* (Bosc) Morgan in Colorado. They documented the effects of FFR fungi on soil physicochemical properties and vegetation, classified FFRs based on their impact and verified that senescent mycelium released nutrients, while its water-repellent properties caused bare zones. Their research incorporated findings from 31 authors and 47 fungal species, though it did not include Bayliss’ 1911 work, a point criticised by Bayliss in 1926. Shantz and Piemeisel noted that FFR effects were species-specific and not directly linked to seasonal rainfall.

A century later, scientific interest in FFRs has diversified. While some efforts have focused on understanding the ecological significance and functionality of *Basidiomycetes* fungi in general (Edwards 1984; Bonanomi et al. 2012; Espeland et al. 2013; Hearst et al. 2013), others have scrutinised their role as phytopathogens in turfgrass (Halisky and Peterson 1970; Terashima et al. 2004; Fidanza 2007a; Miller et al. 2011). More recently, the potential of FFRs for identifying bacterial taxa associated with ectomycorrhizal *Shiro* (Japanese term referring to FFRs) formed by *Tricholomamatsutake* (S. Ito & S. Imai) Singer has been explored for its applications in commercial and forestry settings (Kim et al. 2014; Oh et al. 2016; Oh and Lim 2018). Additionally, “fairy chemicals” produced by FFR fungi have been investigated for their potential as plant hormones to enhance crop productivity, forming a promising area of research (Suzuki et al. 2016; Choi et al. 2017; Kawagishi 2018).

## ﻿Taxonomy of FFR fungi

Recent work by Getzin et al. (2021) has provided important clarification regarding the nomenclature of circular vegetation patterns, distinguishing fungal fairy rings (FFRs) from “fairy circles,” which are not caused by fungi and occur in desert grasslands, such as those in Namibia, South Africa, Angola and parts of Western Australia.

**Figure 4.**
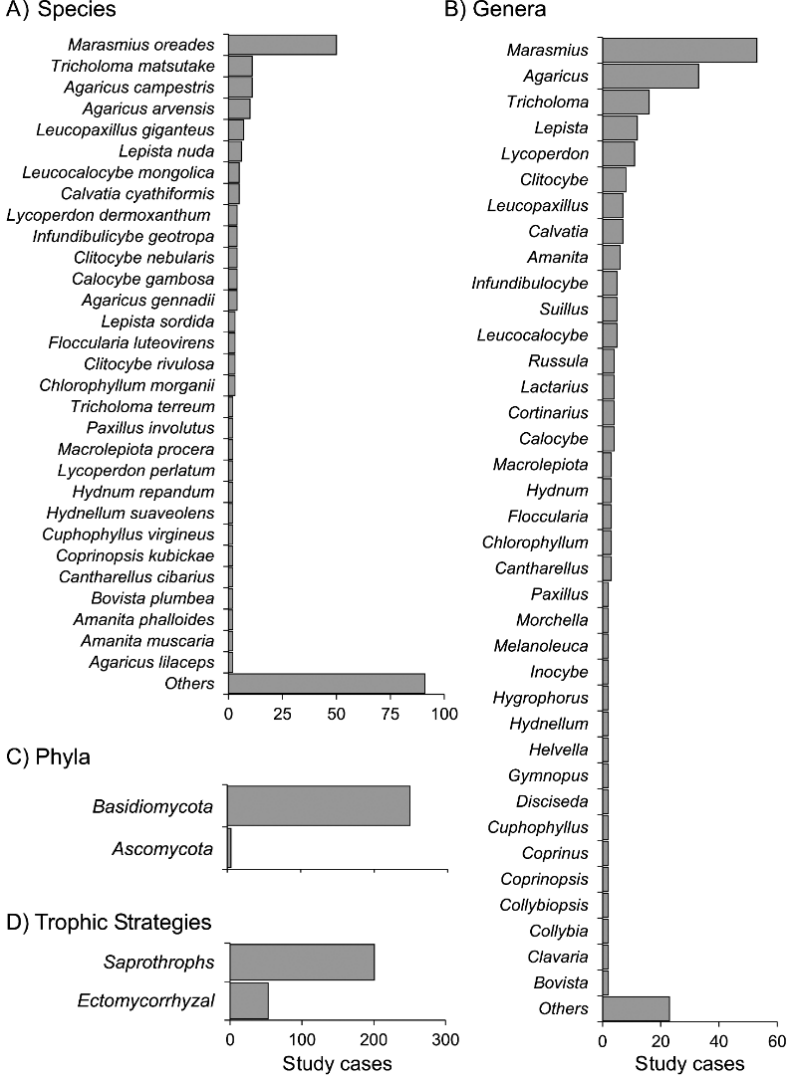
Number of study cases on FFR-forming fungi for different taxonomic levels and ecological functions.

A total of 121 different taxa were recorded forming FFRs in natural environments (Suppl. material 1: table S1), with the majority caused by the activity of *Basidiomycetes*. However, a few instances involving *Ascomycetes* have been documented, including species such as *Helvella* L., *Morchella* Dill. Ex Pers. and *Tuber* P. Micheli ex F.H. Wigg. (Suppl. material 1: table S1). Additionally, many non-sporophore-forming species generate subtle circular patterns that are less conspicuous (Rosa et al. 2020). Amongst *Basidiomycetes*, the most studied species forming FFRs in both natural and managed grasslands include *M.oreades*, *Agaricuscampestris* L. and *Agaricusarvensis* Schaeff. In woodland ecosystems, the ectomycorrhizal fungus *T.matsutake* is the subject of the highest number of studies (Fig. 4).

The functional ecology of FFRs has been an area of significant focus. Gregory (1982) proposed a fundamental classification of FFRs into “free” and “tethered” categories. “Free” FFRs are primarily formed by saprotrophic fungi in grasslands, while “tethered” FFRs are associated with ectomycorrhizal fungi in woodlands. The latter are considered “tethered” due to their reliance on maintaining a symbiotic relationship with host plants. While grasslands predominantly feature FFRs formed by saprobic fungi, woodlands host FFRs generated by both ectomycorrhizal and saprobic species.

A prime example of tethered FFRs in woodland environments is the *Shiro* caused by *T.matsutake*, an ectomycorrhizal symbiont of *Pinusdensiflora* Siebold & Zucc. in Japan (Lian et al. 2006; Peter 2006). Other notable examples include *Clitocybenebularis* (Batsch) P. Kumm., a litter decomposer forming FFRs in *Fagussylvatica* L. forests (Dowson et al. 1989) and *Collybiapinastris* (Kauffman) Mitchel & A.H. Sm., which can expand as a free-living saprotroph in *Piceaabies* (L.) H. Karst forests (Miyamoto and Igarashi 2004). Additional saprobic species that form FFRs on woodland floors include *Infundibulicybegeotropa* (Bull.) Harmaja, *Macrolepiotaprocera* (Scop.) Singer and occasionally *C.gambosa* (Papetti et al. 1999).

Pathogenic fungi also contribute to the formation of fungal fronts resembling FFRs. For instance, in woodlands, the pathogenic *Armillariaostoyae* (Romagn.) Herink creates expansive decay patterns, such as those observed in the forests of Oregon (Ferguson et al. 2003) and in *Pinusmugo* Turra forests in the Alps (Bendel et al. 2006). In agricultural contexts, *Helicobasidium* Pat. species have been documented producing FFR-like patterns in cranberry plantations (Oudemans et al. 2008; Polashock 2009). In Arctic environments, consortia of microbial species, primarily *Ascomycetes*, generate concentric FFRs in mosses (Rosa et al. 2020). Furthermore, the saprotroph *Aspropaxillusgiganteus* (Sowerby) Kühner & Maire has been observed to cause vegetation decay in young pine forests through radial expansion, forming ring-like patterns (Peace 1936).

The observations of Gregory (1982) and the comprehensive report by Toohey (1983) underline the ubiquity of FFR fungi with regular mycelial patterns across diverse ecosystems. The specific species involved in FFR formation are determined by ecological conditions, reflecting the adaptability and ecological roles of these fungi.

## ﻿Ecology of FFRs

### ﻿Occurrence and environmental requirements for FFRs formation

**Figure 5.**
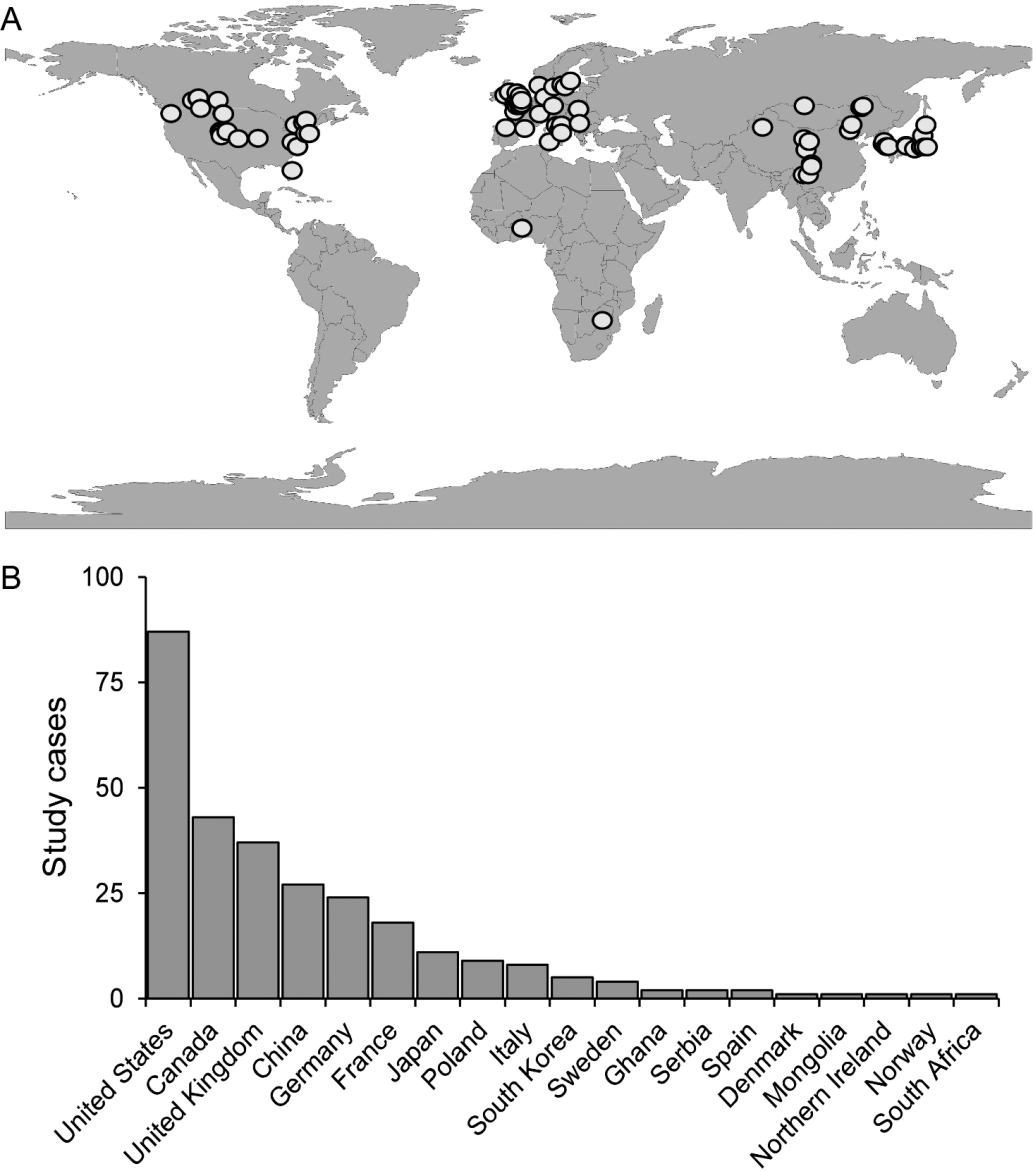
World map distribution of FFR study cases available in the literature (**A**) and their numbers in different countries (**B**).

Fungal Fairy Rings (FFRs) are observed in diverse environments, ranging from woodlands to grasslands and are widespread globally (Gregory 1982; Dowson et al. 1989; Peter 2006; Fig. 5). In woodlands, the formation of FFRs is influenced by the symbiotic relationships with specific plant hosts or the presence of particular types of litter. Conversely, in grasslands, the environmental requirements for FFR fungi depend heavily on the soil-water balance. Bayliss (1911), Shantz and Piemeisel (1917), Hardwick (1978) and Gramss (2005) highlighted that FFRs typically appear in natural grasslands with well-drained soils. Recent studies in the Laramie Basin by Miller and Gongloff (2021) identified that adequate, but not excessive, precipitation is a critical factor for FFR formation. In regions with low precipitation, there is a notable decline in FFR colonies, while higher frequencies of FFRs were documented in north-facing slopes. This aligns with observations by Allegrezza et al. (2022), who reported higher fungal colony densities in sloped areas compared to flat zones, where stagnant water and soil hypoxia inhibit fungal development.

In the Italian Apennines, Allegrezza et al. (2022) found that FFRs are most frequently observed in areas with annual rainfall ranging from 900 to 1300 mm and completely disappear when precipitation exceeds 1700 mm. These findings suggest that geomorphological and hydrological conditions significantly influence the spread of Basidiomycete colonies.

### ﻿Influence of altitude and temperature on FFRs distribution

While precipitation patterns largely determine the presence of FFR fungi, altitude and temperature are less restrictive. In the Italian Apennines, high frequencies of FFR colonies were recorded at altitudes between 500 and 2200 m above sea level, with mean annual temperatures ranging from 3 to 13 °C. FFRs are also documented in high-altitude grasslands, such as the Tibetan Plateau, where *Floccularialuteovirens* (Alb. & Schwein.) Pouzar forms rings at around 3800 m above sea level, with a mean annual temperature of -3.9 °C (Wang et al. 2005).

Despite these extremes, many species have wide altitudinal ranges, such as *M.oreades*, observed from sea level (Molliard 1910) to 1730 m above sea level (Norstadt et al. 1973). Certain species, like *Chlorophyllummolybdites* (G. Mey.) Massee is confined to tropical climates (Reid and Eicker 1991), highlighting the variability in ecological niches occupied by FFR-forming fungi.

### ﻿Soil type and nutrient influence on FFRs development

Soil type is another key factor in FFR formation. Miller and Gongloff (2021) noted that, amongst 16 soil types studied, FFRs occurred in eight types belonging to Aridisols and Mollisols. Taxa such as *Agaricusbraendlei* L.A. Parra & M.M. Gómez, *Agaricuslilaceps* Zeller, *Calvatia* Fr., *Discisedacandida* (Schwein.) Lloyd and *Geastrum* Pers. prefer Aridisols, while *M.oreades* and *Bovistaplumbea* thrive in Mollisols, likely due to lower levels of dissolved salts from precipitation.

Nitrogen availability also affects FFR density in grasslands, as observed in studies linking FFR prevalence to cattle manure (Cosby 1960; Hardwick and Heard 1978). Excessive nitrogen, however, is detrimental to fungal growth. High nitrogen levels impair mycelial health (Kües and Liu 2000) and enzymatic activity (Kachlishvili et al. 2006). In agricultural systems with high nitrate release from soil tillage, FFRs are rare, with only one case reported in a barley field previously occupied by grassland (Shantz and Piemeisel 1917). Phytopathological research further confirms that excessive inorganic nitrogen limits the spread of FFR-forming fungi like *M.oreades* in gardens and turfs (Drew Smith 1957).

### ﻿Biogeographic and ecological factors modulating FFRs formation

In the Italian Apennines, biogeographic surveys reveal that FFRs are more prevalent at higher altitudes, likely due to reduced grazing pressure, as these grasslands are accessible to herbivores for only a few months annually (Allegrezza et al. 2022). Interestingly, some fungi benefit from the presence of herbivores. For instance, *A.arvensis* thrives in horse-grazed grasslands in the Rogedano Mountain, Marche, Italy, where grazing is restricted to wild horses and a few wild herbivores such as deer (Bonanomi et al. 2012; Bonanomi et al. 2013).

### ﻿Additional factors favouring FFRs formation

Undisturbed environments with moderate nutrient levels and abundant decomposing organic matter are conducive to FFR formation. Although there is no direct evidence linking specific nutrient levels in grasslands to particular FFR fungi, stable grasslands often support *Basidiomycetes*, which serve as bioindicators of environmental disturbance (Arnolds 1992; Griffith et al. 2002; Durall et al. 2005).

Specific conditions, such as litter type and the absence of competitors, can also promote FFR development. For example, *C.nebularis* specialises in degrading broadleaf litter in woodlands, but cannot thrive in grasslands due to the unsuitability of grass litter. Similarly, *C.gambosa* (Saint George’s mushroom) prefers to grow beneath *Rosaceae* Juss. plants such as *Prunusspinosa* L. and *Rubusulmifolius* Schott or under old plantations of *Sorbus* L., *Malus* Mill. and *Pyrus* L. Although the reasons for this preference remain unclear, it is speculated that the lack of ectomycorrhizal symbiosis in most *Rosaceae* plants favours the growth of saprotrophic fungi (Gadgil and Gadgil 1971).

## ﻿FFRs classification

**Figure 6.**
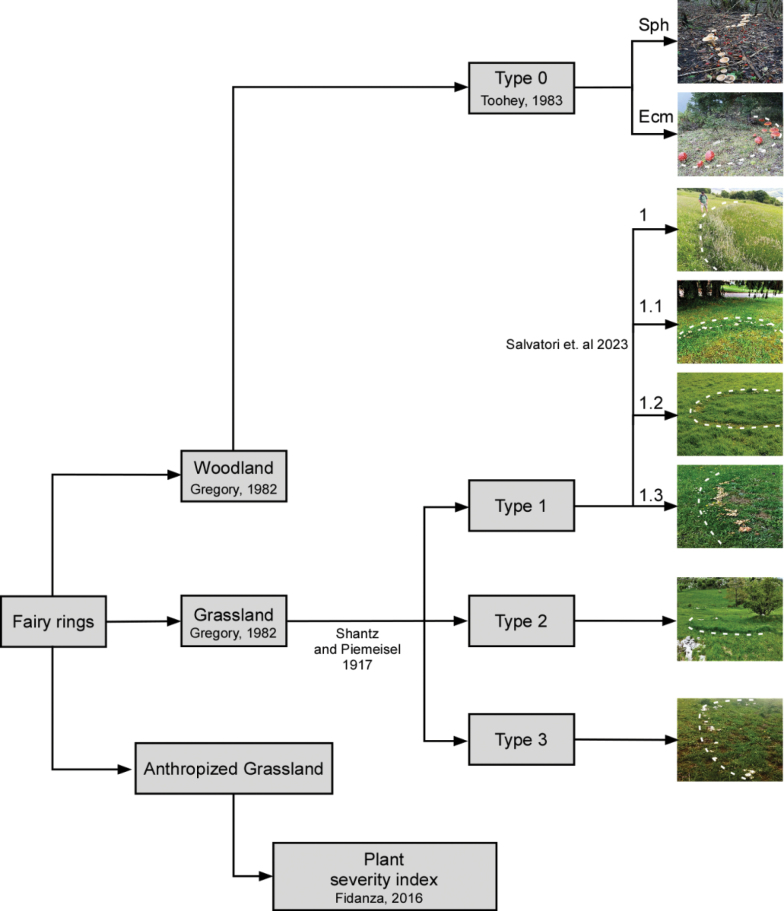
FFRs classification according to habitats and effects on vegetation. Sph and Ecm abbreviations in the diagram refers to FFRs formed by saprotroph and ectomycorrhizal fungi, respectively.

The detection and study of FFRs fungi in grasslands have historically been facilitated by their visible effects on vegetation or the presence of sporophores in circular arrangements, which trace dominant fungal fronts in the soil. The classification of FFRs, based on their effects on vegetation, has proven instrumental in understanding the interactions between fungal mycelium and soil biota (Fig. 6). In semi-natural grasslands, Shantz and Piemeisel (1917) introduced the first classification system, which remains widely used to delineate FFRs by their impacts on grass cover. This system identifies three types of FFRs: Type 1 FFRs exhibit two distinct vegetation zones; a narrow external belt of barren soil or inhibited vegetation and an internal belt of enhanced plant growth, marked by a darker green colour (Fig. 1A). Type 2 FFRs are characterised by a single belt of darker vegetation without an external necrotic zone. In contrast, Type 3 FFRs cause no discernible changes to vegetation and are revealed only seasonally through sporophore emergence.

Building on this foundational work, Toohey (1983) expanded the classification to include ectomycorrhizal and saprobic FFRs in woodlands, where sparse vegetation prevents visual assessment of effects. These were designated as Type 0 FFRs, which exhibit regular patterns of extension in woodland soils (Fig. 6). More recently, Salvatori et al. (2023) refined the classification of Type 1 FFRs into three subtypes. Type 1.1 features an external belt of stimulated vegetation smaller than the internal stimulated belt, separated by a barren zone. In Type 1.2, the external stimulation zone is larger than the internal one. Type 1.3 lacks stimulation and is identified solely by a barren soil belt. However, this updated classification omits tripartite FFRs, such as those observed in *A.arvensis* (Edwards 1984, 1988) and *M.oreades* (Molliard 1910; Gramss 2005), which exhibit Type 1 effects on the outer edge and Type 2 effects in concentric inner rings.

FFRs have also been classified by their impacts on turfs and gardens, employing an index to quantify the severity of fungal fronts as phytopathological issues (Fidanza et al. 2016). Type 3 FFRs are assigned an index value of 1, reflecting their lack of symptoms on grass cover. Type 2 FFRs are graded with indices from 2 to 4, depending on the intensity of their stimulatory effects on vegetation. Transitional forms from Type 2 to Type 1 are indexed as 5 or 6, depending on whether stimulation and slight wilting or moderate wilting dominate the external edges. Finally, Type 1 FFRs are indexed from 7 to 9, correlating with the progression from moderate to severe necrosis or complete vegetation death.

An alternative classification system considers the depth of mycelial spread in the soil. Couch (1995) and, later, Fidanza (2007a) distinguished FFR mycelial mats based on their association with grass leaf litter (leptophilic) or deeper soil horizons (edaphic). Although developed for FFRs in turfs, this classification applies broadly across natural and artificial ecosystems.

Regarding FFR morphology, grassland FFRs are particularly well-studied due to their permanence and distinct patterns, such as ribbons, arcs and rotors. In contrast, limited research exists on FFR shapes in woodlands, especially ectomycorrhizal forms. These likely follow analogous patterns, but on different scales, influenced by the forest floor’s discontinuous environment, higher density of competing fungal species and multiple mycelial starting points from symbiotic root tips.

Parker-Rhodes (1955) first hypothesised that FFR genesis could be likened to waves from a droplet in a pond, with the circular shape maintained in unperturbed microenvironments. Disruptions, such as soil obstacles or perturbations, (Davidson et al. 1996a; Davidson et al. 1996b) may fragment this circularity into arcs or other shapes (Shantz and Piemeisel 1917; Parker-Rhodes 1955; Stevenson and Thompson 1976; Karst et al. 2016). Geomorphological features also influence FFR shapes. Miller and Gongloff (2021) and Allegrezza et al. (2022) observed arcs as the most common FFR forms, followed by rings, with rotor-like formations being rare. They proposed that arcs and rotors form in sloped areas due to the downward washing of autotoxic compounds, such as self-DNA, facilitating fungal growth at the colony’s lower edge. Conversely, rings predominate in flat terrains (Stevenson and Thompson 1976; Karst et al. 2016).

Rotor-like FFRs, amongst the most intriguing forms, are created when the terminal tips of an arc introflect, producing a curly pattern. Karst et al. (2016) suggested that these result from portions of mycelium escaping control at the leading edges of arcs. Similarly, spirals form through asymmetric introflection, paralleling clonal plant patterns like Archimedean spirals in desert environments (Fernandez-Oto et al. 2019).

Other complex shapes, such as “papillon” or “moustaches”, arise from coalescing mycelial mats. Parker-Rhodes (1955) and Stevenson and Thompson (1976) modelled these shapes mathematically, examining the kinetics, shapes and interactions of fungal fronts. Their models revealed three potential outcomes when advancing mycelial fronts intersect: indifference, where both continue to grow and cross; unilateral extinction, where one is obliterated; and bilateral extinction, where both disappear at the intersection, yielding coalescence-based FFR shapes.

## ﻿Spatial dynamics of FFRs

### ﻿FFR genesis and growth

A distinctive feature of fungal fronts is the regular arrangement of their mycelial mats, originating from a single point and spreading centrifugally, akin to fungal growth observed in Petri dish cultures. These fronts may arise from germinating spores or vegetative expansion of mycelium fragments. Studies on *M.oreades* in Norwegian sandy dunes provided evidence that most fungal fronts (~ 90%) are generated by spore germination rather than mycelium fragmentation, as indicated by their distinct genetic structures (Abesha et al. 2003). In rare cases, fungal fronts form vegetatively, involving spatial advancement and intraspecific coalescence. Formation of FFR colonies with more than one genet can also be observed (Mallett and Harrison 1988), as observed in pathogenic species like *A.ostoyae* (Legrand et al. 1996; Worrall et al. 2004) and ectomycorrhizal fungi such as *T.matsutake*, where up to four genets can form the same *Shiro* (Lian et al. 2006; Peter 2006).

The expansion of fungal mycelium in soil is driven by various factors, including precipitation and temperature (Moore et al. 2008), soil resource distribution (Ritz 1995), microbial competition, parasitism and chemical interference (Dix 2012). Self-regulation mechanisms, such as the release of inhibitory substances, also play a critical role (Keller et al. 2005; Mazzoleni et al. 2015a; Mazzoleni et al. 2015b; Salvatori et al. 2023). Actively growing hyphae colonise outer soil regions in search of organic matter (Mathur 1970; Miyamoto and Igarashi 2004; Baldrian 2008; Dix 2012) (Fig. 1A, F) or plant hosts as in the case of fronts formed by pathogenic fungi (Shaw III 1980; Anselmi and Minerbi 1988; Pukkala et al. 2005), releasing enzymes that degrade organic material and assimilating nutrients from newly colonized areas (Šnajdr et al. 2011; Rodrigues et al. 2015). Fungal fronts form regular patterns only when resources are homogeneously distributed in soil. Otherwise, mycelial growth becomes patchy, reflecting resource availability (Bayliss 1911; Shantz and Piemeisel 1917; Mathur 1970; Dowson et al. 1989; Griffith and Roderick 2008).

As new mycelium explores the soil, the internal regions of fungal fronts are conditioned by senescent mycelial residues, rendering these areas unsuitable for recolonisation by younger hyphae, which remain confined to the fungal front’s margins (Mazzoleni et al. 2015b; Álvarez-García et al. 2020). This phenomenon, observed across diverse fungi under laboratory conditions, underpins a central question raised by Bayliss (1911): why do fungal fronts form rings rather than dish-like colonies?

Historically, the nutrient-based hypothesis posited that nutrient depletion in the internal zones of fungal fronts prevents recolonisation (Wollaston 1807; Way 1847; Lawes et al. 1883). However, this explanation lacked empirical support. Way (1847) and Schreiner and Reed (1907) endorsed De Candolle’s excretory theory (1830–1832), suggesting that recolonisation is inhibited by species-specific excretory by-products. Experimental evidence supporting unidirectional fungal growth was provided by a sod inversion study, where *C.nebularis* failed to grow in soil it had previously conditioned (Dowson et al. 1989).

The inability of fungal fronts to recolonise inner areas is now understood within the broader framework of biological pattern formation. Ring-like patterns are also observed in plant tussock rings (Bonanomi et al. 2014; Cartenì et al. 2016). Mazzoleni and co-workers (2015a, 2015b) suggested that patterns of circular growth, both for fungi and plants, can be explained by self-DNA autotoxicity, as decomposition of senescent organic matter releases chemical compounds with specific detrimental effect on the species that produced it. The presence of self-produced waste products impairs the ability of vegetative organisms to recolonize the inner portions of the colony despite resources availability. Recently, the self-inhibitory hypothesis mediated by self-DNA was reproduced by means of mathematical modelling. In the model, the authors successfully reproduced the FFRs circular shapes, demonstrating that autotoxicity provides an explanatory mechanism of fungal front dynamics in the soil space (Salvatori et al. 2023).

In sloped terrains, fungal fronts often develop into arc-like patterns, with degeneration observed downslope due to leaching of water-soluble self-DNA (Miller and Gongloff 2021; Allegrezza et al. 2022). This phenomenon results in a transition from closed rings, typical in flat areas, to open arcs, as fungal colonies retreat uphill leaving behind a self-toxicity tail.

### ﻿Growth rates, ages and depth

Both ecologists and plant pathologists commonly estimate the annual growth rate of fungal fronts (FFRs) by tagging the external edge of the zones where active mycelium is present and monitoring the subsequent metrical advancement over the course of a vegetative season. The rate of mycelial expansion depends on several factors, including fungal species, seasonality and vegetation type (Ramsbottom 1926; Pugh 1980; Toohey 1983). In unfavourable conditions, the widening of fungal front regions (FFRs) can be temporarily halted for several years, suggesting that age calculations, based on previous expansions, may have been overestimated (Ramsbottom 1926). Nevertheless, this remains one of the most reliable observational methods to assess colony growth rate and age.

More recently, Miller and Gongloff (2023) estimated the size and age of 304 FFRs in the Laramie Basin by comparing aerial photos taken from 1947 to 2022. Their approach, which incorporated field estimates of the FFR-forming fungi’s specificity, provided the most comprehensive insight into the colonisation dynamics of FFRs in grassland ecosystems.

**Figure 7.**
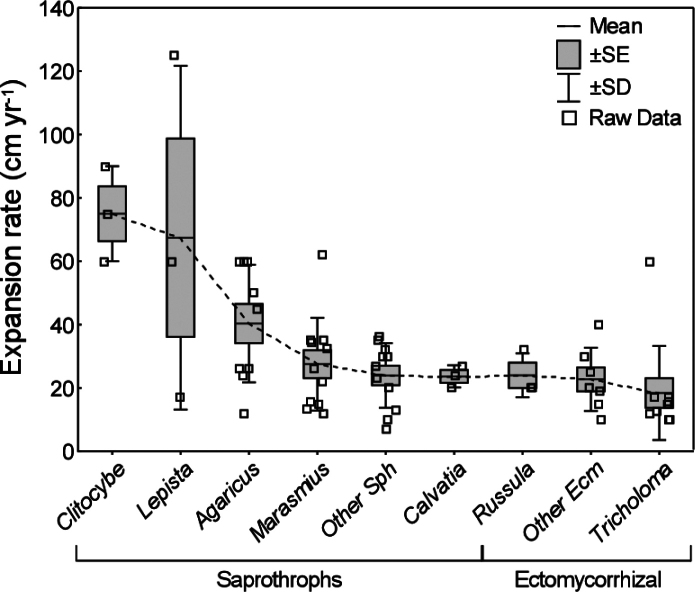
Annual expansion rate of fungal fronts from different FFR-forming fungi (60 observations) divided by genera and ecological groups. Dashed line connects mean points. Sph and Ecm abbreviation refers to FFRs formed by saprotroph and ectomycorrhizal fungi, respectively.

The growth rates of FFRs vary considerably across different fungal taxa (Fig. 7). Species with the highest growth rates are typically those within the *Clitocybe* (Fr.) Staude genus, with average rates of approximately 75 cm per year (Toohey 1983; Dowson et al. 1989). For example, *Lepista* (Fr.) W.G. Sm. species exhibit particularly high growth rates, with *Lepistasordida* (Schumach.) Singer showing an annual growth of 125 cm in turf environments (Terashima et al. 2004), though the average growth rate for this genus is closer to 60 cm per year. Similarly, fungi in the *Agaricus* L. genus have average growth rates of around 55 cm per year (Edwards 1984; Bonanomi et al. 2012). The *Marasmius* Fr. genus shows a broader growth range, from 7 to 39 cm per year (Cosby 1960; Burnett and Evans 1966; Ingold 1974; Hardwick and Heard 1978; Dickinson 1979). Slower-growing taxa include ectomycorrhizal species, such as *Russula* Pers. and *Tricholoma* (Fr.) Staude, with average growth rates of 22 cm per year and 17 cm per year, respectively (Ohara and Hamada 1967; Lian et al. 2006; Kataoka et al. 2012; Narimatsu et al. 2015).

The methodology for estimating growth rates, in combination with the diameter of the FFRs, has enabled the estimation of fungal front ages. In French grasslands, large FFRs of *I.geotropa*, with a diameter of 800 m, were estimated to be around 700 years old (Drew Smith 1957; Gregory 1982). Similarly, a FFR with a diameter of 137 m and an average growth rate of 60 cm per year would be approximately 100 years old (Allegrezza et al. 2022). Seasonal extension methods, combined with rhizomorph log colonisation rates, have also been used to assess the growth rates of pathogenic *Basidiomycetes*, such as *Armillariagallica* Marxm. & Romagn. With a growth rate of around 20 cm per year and a minimum expansion of 15 hectares, these fungi were estimated to be 1,500 years old, with an estimated biomass of 10,000 kg (Smith et al. 1992). Similar methods have been applied to other fungal species, including *A.ostoyae*. In Oregon, this species formed an expansive front of 890 hectares, estimated to be 2,400 years old, while a similar front in Switzerland, covering 500 hectares, was approximately 1,000 years old (Ferguson et al. 2003; Bendel et al. 2006). These fungal fronts are amongst the largest living organisms on Earth and their longevity is remarkable, particularly considering that they belong to microbiological life forms. The long lifespan of *Basidiomycetes* may result from a poorly-understood mechanism that preserves genetic integrity and prevents the accumulation of disadvantageous mutations during cell division (Hiltunen et al. 2019; Hiltunen et al. 2021).

While tracking the radial expansion of FFRs is relatively straightforward, representing mycelial development along the soil horizon is more challenging and has been documented only in a few studies (Shantz and Piemeisel 1917; Norstadt et al. 1973; Edwards 1984). Soil trenching has provided valuable insights into how mycelial mats of FFRs are distributed along the soil horizon during the vegetative season and at varying soil depths. The depth of mycelial mats varies amongst FFRs formed by different species (Suppl. material 1). For instance, *Lycoperdoncurtisii* Berk., *L.sordida* and *Holocotylondermoxanthum* (Vittad.) R.L. Zhao & J.X. Li form dense mycelial mats that extend up to 4 cm deep (Terashima et al. 2004). In contrast, species such as *A.campestris*, *T.matsutake* and *A.arvensis* have mycelial mats that extend to greater depths up to 22 cm, 15 cm and 10 cm, respectively (Edwards 1984; Bonanomi et al. 2012; Kataoka et al. 2012).

## ﻿FFRs as ecosystem engineers

### ﻿Impacts on soil physical and chemical properties

**Figure 8.**
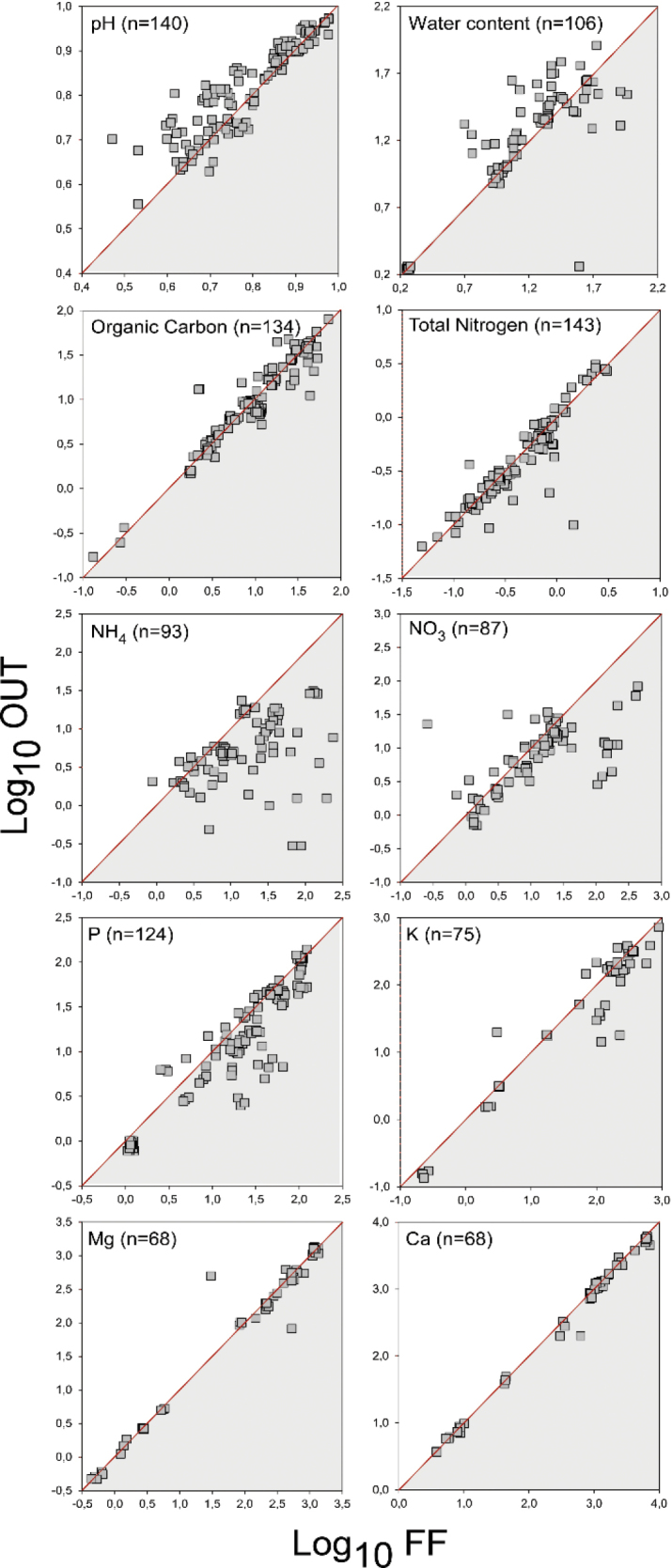
Chemical analysis of soil affected by fungal fronts of FFRs. Data from a total of 180 published FFRs and additional data from 13 FFRs of *Calocybegambosa*, *Agaricusarvensis*, and *Agaricuscrocodilinus* in Italian grassland/woodland. Data were collected as chemical parameters in the fungal front and outer areas with a high density of mycelial mats and soil external to FRs, respectively. Collected data were converted to mg kg^-1^ (ammonia, nitrates, phosphorous, potassium, magnesium, calcium) or % (total organic carbon, total nitrogen, pH, water content), Data were log-transformed to reduce dimensionality. Values of n represent the number of FFRs where soil chemical variables were studied.

As with other filamentous fungi, the fungal fronts of FFRs fungi form at the outermost periphery of the mycelial mats, where hyphae extend their apices to colonise organic matter. Once the target is reached, intense sub-apical branching fills all available space in the substrate. In *M.oreades*, young vegetative hyphae at the outer edge of the mycelial mats secrete elevated levels of extracellular laccases, which catalyse the oxidation of organic matter (Mathur 1970; Yaropolov et al. 1994; Gramss et al. 2005; Griffith and Roderick 2008). This process breaks down compounds such as lignin and cellulose, which, in turn, lead to soil denitrification and the release of CO_2_, resulting in soil acidification due to the reduction of pH (Fig. 8) (Gramss et al. 2005; Bonanomi et al. 2012; Zotti et al. 2021).

Concurrently, in the bulk of the soil affected by FFRs, water content decreases (Fig. 8), a side effect of hydrophobin secretion (Sietsma et al. 1995; York and Canaway 2000; Fidanza 2007b). Hydrophobins in FFR fungi likely serve multiple functions (Wösten 2001), including regulation of water within the substrate (Lugones et al. 1998), converting hydrophobic substrates into hydrophilic ones (Wösten and de Vocht 2000), providing protection from drying and air exposure (Wösten 2001), acting as mechanical barriers against pathogen attacks and aiding in correct sporophore development (De Groot et al. 1996; De Groot et al. 1999). These hydrophobins also regulate water content, making the secreted chemicals more effective (Lebeau and Hawn 1961; Blenis et al. 2004; Caspar and Spiteller 2015), which benefits FFR fungi in their biological activity. Similarly, some ectomycorrhizal mats also produce hydrophobic mycelial aggregates, suggesting that both symbiotic and saprobic fungi share similar strategies (Griffiths et al. 1994; Koo et al. 2003; Koo et al. 2009).

Significant changes occur in the levels of ammonia and nitrates in the soil affected by FFRs (Shantz and Piemeisel 1917; Fisher 1977; Edwards 1988; Fidanza 2007b; Espeland et al. 2013; Yang et al. 2019). The mycelial mats absorb decomposed material from the outer edge of the colony, releasing excess intracellular nitrogen in the form of ammonium (De Groot et al. 1998; Moore 2003). Once released, ammonium is converted into nitrates by the activity of associated bacteria (Shantz and Piemeisel 1917; Fisher 1977; Edwards 1984; Zotti, Bonanomi et al. 2021). These nitrates undergo denitrification and are released as gaseous nitrogen (Knowles 1982). However, basidiomycete mycelium cannot utilise nitrogen in this gaseous form (Deacon 2013) and it is, instead, absorbed by plants. In the case of ectomycorrhizal fungi, high ammonium concentrations have been observed in *T.matsutake* (Kim et al. 2013) and mycelial mats of *Hysterangium* spp. Vittad. (Griffiths et al. 1994; Kluber et al. 2010; Trappe et al. 2012), but not in nitrates, leading to the hypothesis that nitrogen compounds released by decomposition are directly passed to the plant symbionts without undergoing bacterial transformation.

Parallel to the release of ammonium and nitrates, several FFR fungi have shown consistent enrichment of phosphorus in the soil (Fisher 1977; Edwards 1984; Fidanza 2007b; Yang et al. 2018a, 2018b). Phosphorus is essential for many biological processes and its high enrichment in FFR soil may be due to its decomplexation from organic matter (Jennings 1989). It may also play a role as a co-factor in the selective decomposition of recalcitrant compounds (Tripathi and Yadav 1991) and could be translocated to the colony edge for nutritional purposes, similar to rhizomorph-forming fungi (Wells and Boddy 1995; Wells et al. 1998). Recent studies have shown that the high levels of phosphorus in FFR soils are due to its available forms, as measured by Olsen P methodologies, indicating that it is released through organic matter decomposition (Gramss et al. 2005; Bonanomi et al. 2014; Yang et al. 2019; Zotti et al. 2021). However, slightly decreased phosphorus levels have occasionally been observed within FFRs, suggesting that a portion of phosphorus is sequestrated in the active mycelium (Fisher 1977; Edwards 1984; Gramss et al. 2005). Phosphorus is used in sporophore formation, a high-energy-demanding process (Kües and Liu 2000) and is, therefore, reabsorbed from the soil during fruit-body emergence (Fisher 1977).

Mycelial activities also lead to the accumulation of higher levels of potassium, magnesium and calcium, resulting from the solubilisation of these elements during organic matter decomposition (Gramss et al. 2005). While the specific dynamics of these elements in FFR fungi are not well understood, their contribution to fungal nutrition is acknowledged (Deacon 2013).

Lastly, iron levels increase with the passage of FFR mycelial mats. This has been observed in *C.gambosa* (Zotti et al. 2021) and *M.oreades* (Gramss et al. 2005), where iron concentrations doubled. It is likely that iron sequestration occurs due to the high demand for the element in carbohydrate consumption and homeostasis. Additionally, iron may be gathered from the external soil environment through the production of siderophores (Renshaw et al. 2002).

### ﻿Effects on higher plants

The impact of FFRs on grassland ecosystems and vegetation can be complex and varied. In some cases, the mycelium can damage the grass cover, while in others, it can stimulate lush plant growth. Both outcomes can be observed in many FFRs and, when these fungi spread at the edges of forested and grass-dominated environments, their influence on vegetation is evident, even in ectomycorrhizal species (Toohey 1983). Given the dual nature of these effects, this section first provides an overall description of the influence of FFR fungi on vegetation, then divides the discussion into two subsections. This structure aims to clarify the mechanisms behind both the detrimental and stimulating effects of FFR fungi on plant growth, while also exploring the ecological implications of advancing mycelial mats on plant communities.

#### ﻿Detrimental effect of FFRs on plants

The detrimental effects of FFRs on plant growth have been extensively studied. Shantz and Piemeisel (1917) hypothesised that the death or growth impairment of plants is often due to drought caused by the hydrophobic nature of mycelial mats. This hypothesis is based on the role of hydrophobins in the fungal mycelium, which create hydrophobic conditions in the soil, reducing water availability for plants and inducing drought stress (Sietsma et al. 1995; Carminati et al. 2009). Evidence supporting this idea includes the persistence of water droplets on FFR-affected soil, which can take minutes to be absorbed (Fidanza 2007b; Bonanomi et al. 2012; Zotti et al. 2021). The lack of water in the soil causes root systems to shrink, creating air gaps avoiding root-soil contact (Carminati et al. 2009). This effect has been observed in species-rich grasslands, where grasses with thin fasciculate root systems are often killed by the mycelial mats, while tap-rooted plants can survive by accessing deeper water reserves (Bonanomi et al. 2012; Zotti et al. 2020).

In certain environments, such as gardens, lawns, golf courses and agricultural fields, the presence of FFR fungi can lead to patches of dead vegetation, often observed during dry periods (Filer 1965; Fidanza 2007a; Fidanza 2017). The effect is considered negative, leading to a massive research effort to understand the best eradication method of FFR fungi (Halisky and Peterson 1970; Smith and Rupps 1978; Miller Jr 2010; Miller et al. 2012). Interestingly, the formation of these dead belts can be specific to the fungal species and its soil colonisation strategy. For example, in Type 2 *C.cyathiformis*FFRs, mycelial mats are sparsely distributed, resulting in stimulation of grass cover. In contrast, the dense aggregation of *A.praerimosus* mycelium leads to the decay of the existing plant community (Shantz and Piemeisel 1917). Furthermore, the incidence of plant death due to drought in FFRs is well-documented in several studies (Drew Smith 1957; Cosby 1960; Norstadt et al. 1973; Ingold 1974; Fisher 1977; Hardwick and Heard 1978; Toohey 1983; Couch 1995; Blenis et al. 1997; Fidanza 2007a; Fidanza 2007b; Fidanza et al. 2007).

Apart from hydrophobicity, other factors contribute to the formation of dead vegetation belts. Water extracts from mycelial mats of *M.oreades* have been shown to possess phytotoxic properties that affect plant growth (Bayliss 1911). These extracts can alter root architecture and damage fine roots (Elliott 1926). In field conditions, *M.oreades* can also produce phytotoxic compounds, including cyanides, which further harm plants (Lebeau and Hawn 1961; Blenis et al. 2004). Caspar and Spiteller (2015) proposed that the release of cyanuric compounds by FFR fungi serves as a defensive mechanism. In addition, *M.oreades* hyphae have been observed to penetrate cortical tissues and kill epidermal cells of plants, forming a dense mycelial mantle around the root systems of infected plants (Filer 1965).

Modelling studies of FFR effects on plants Salvatori et al. (2023) have shown that hydrophobicity is the primary factor leading to plant death, while the parasitic or toxic behaviour of the fungus may act as a secondary factor. However, it is also possible that hydrophobins contribute to the hydrophilisation of plant cell cortex during pathogeneses (Wösten 2001) or that drought-induced damage increases the plants’ susceptibility to the fungus, leading to the decomposition of affected root cells. Further studies are needed to better understand the precise mechanisms underlying plant death caused by FFRs.

On an ecosystem scale, FFRs significantly influence grasslands and can even affect the growth of young trees in forests (Peace 1936). The mycelium creates a biological disturbance that kills dominant plant species in the grassland, leading to a shift in community composition (Cosby 1960; Hardwick and Heard 1978; Bonanomi et al. 2012; Zotti et al. 2020). This disturbance opens up ecological niches, which are quickly colonised by short-lived, fast-growing plant species. However, the effect is temporary and the original plant community typically recovers over time (Bonanomi et al. 2012; Zotti et al. 2020). These changes in plant communities are more pronounced in Type 1 FFRs, which cause significant plant death or growth impairment (Shantz and Piemeisel 1917). The impact of FFRs on plant communities may be particularly strong during dry periods when the mycelial mats exacerbate drought conditions.

Management practices such as mowing or grazing can influence the activity of FFR fungi and, consequently, the alteration of plant communities (Cosby 1960; Hardwick and Heard 1978). In the long term, the changes induced by fungal-driven disturbances can lead to an enrichment of plant species in grasslands, as normally outcompeted plants are given an opportunity to thrive. Bonanomi et al. (2012) suggested that FFR fungi could function as ecosystem engineers, modulating species co-existence in Mediterranean grasslands, a concept later supported by multi-kingdom scale observations (Zotti et al. 2020; Zotti et al. 2021).

#### ﻿Stimulating effect of FFRs on plants

For more than two centuries, scientists have thought that the natural fertilisation effect of fungi was the primary reason for the greener belts of vegetation associated with FFRs. Recent studies, however, have provided evidence that FFR fungi can influence plant growth through several mechanisms beyond simple nutrient enrichment (Table 1).

**Table 1.** Bibliographic collection and focus of studies and species regarding FFR from 1917 to nowadays. FFRs are defined according to classification in Fig. 6. Abbreviation: P: Plants; F: Fungi; B: Bacteria, NA: not available.

Species	Type	Community effects	Stimulated Bands	Necrotic Bands	Focus	Site	References
*Agaricuspraerimosus*	T1	Favour opportunistic species	Nutrients	Hydrophobic soil	P	USA, grassland	Shantz and Piemeisel 1917
*Calvatiacyathiformis*	T2	No changes	Nutrients	NA	P	USA, grassland	Shantz and Piemeisel 1917
*Marasmiusoreades*	T1	NA	NA	Cyanides	P	USA, lab.	Elliott 1926
*Marasmiusoreades*	T1	Changes depend by manging practices.	Nutrients	Hydrophobic soil	P	USA, grassland	Cosby 1960
*Marasmiusoreades*	T1	NA	NA	Hydrophobic soil, cyanides	P	Canada, lab.	Lebeau and Hawn 1963
*Marasmiusoreades*	T1	NA	NA	Pathogenic behaviour	P	USA, lab.	Filer 1965
*Tricholomamatsutake*	T0	Decrease in bacterial diversity	NA	NA	B	Japan, woodland	Ohara and Hamada 1967
*Marasmiusoreades*	T1	NA	NA	Hydrophobic soil	P, B	USA, turfs	Norstadt et al. 1973b
*Marasmiusoreades*	T1	NA	NA	Nutritive impairment	P	Canada, turf	Fisher 1977
*Marasmiusoreades*	T1	Favour opportunistic species	NA	NA	P	UK, grassland	Hardwick and Heard 1978
*Marasmiusoreades*	T1	Changes in plants	Nutrients	Hydrophobic soil, Nutritive impairment	P	UK, grassland	Edwards 1984; Edwards 1988
*Marasmiusoreades*	T1	NA	NA	Hydrophobic soil, Nutritive impairment	P	UK, grassland	Ayer et al. 1989
*Marasmiusoreades*	T1	NA	NA	Toxic compounds	P	UK, grassland	Sutton 1990 1989
*Marasmiusoreades*	T1	NA	NA	Cyanides	P, F	Canada, turf/lab.	Blenis et al. 2004
*Lycoperdoncurtisii*	T1	NA	NA	Pathogenic behaviour	P	Japan, turfs/lab	Terashima et al. 2004
*Lycoperdondermoxantum*	T1	NA	NA	Pathogenic behaviour	P	Japan, turfs/lab	Terashima et al. 2004
*Marasmiusoreades*	T1	NA	NA	Hydrophobic soil, Nutritive impairment	P	Germany, turfs	Gramss et al. 2005
*Tricholomamatsutake*	T0	ECM species excluded	NA	NA	F	Japan, woodland	Lian et al. 2006
*Agaricuscampestris*	T1	NA	NA	Over enrichment of NH_4_, H_2_S.	P	USA, grassland	Fidanza 2007b
*Lepistasordida*	T1	NA	Fairy chemicals	NA	P	Japan, Lab.	Choi et al. 2010a
*Lepistasordida*	T1	NA	NA	Fungal inhibitor	P	Japan, Lab.	Choi et al. 2010b
*Agaricuscampestris*	T2	NA	Nutrients	NA	P	China grassland	Xu et al. 2011
*Tricholomamatsutake*	T0	Decrease in bacterial diversity	NA	NA	B	Japan, woodland	Kataoka et al. 2012
*Agaricuscampestris*	T1	Favour annual plants	Empty niche, microbiome	Hydrophobic soil, cyanides	P, F, B	Italy, Grassland/Lab.	Bonanomi et al. 2012
*Tricholomamatsutake*	T0	Community simplification	NA	NA	F	South Korea, woodland	Kim et al. 2013
*Clitocybenebularis*	T0	Selective inhibition	NA	NA	F	UK, woodland	Hearst et al. 2013
*Agaricuslilaceps*	T2	NA	Nutrients, microbiome	NA	P, B	USA, grassland	Caesar et al. 2013
*Tricholomamatsutake*	T0	No change	NA	NA	B	South Korea, woodland	Kim et al. 2014
*Tricholomamatsutake*	T0	Change in bacteria, no change in fungi	NA	NA	F, B	South Korea, woodland	Oh et al. 2016
*Floccularialuteovirens*	NA	Community simplification	NA	NA	F, B	China grassland	Xing et al. 2018
*Agaricusgennadii*	T2	No change	Change in N:P ratios	NA	P	China grassland	Yang et al. 2018a
*Agaricusgennadii*	T2	Increase in bacterial diversity	Nutrients	NA	P, B	China grassland	Yang et al. 2018b
*Agaricusgennadii*	T2	No change	Sensitivity to Q10 values	NA	P	China grassland	Yang et al. 2018c
*Agaricusgennadii*	T2	No change	Nutrients	NA	P, B	China grassland	Yang et al. 2019
*Agaricuscampestris*	T2	No change	Nutrients	NA	P, B	China grassland	Yang et al. 2019
*Agaricusarvensis*	T1	Community simplification, opportunistic species	Microbiome	Toxic compounds	P, F, B	Italy, Grassland	Zotti et al. 2020
Multispecies	T2	Increase of fungal diversity	NA	NA	F	Spain, grassland	Marì et al. 2021
*Calocybegambosa*	T1	Community simplification, copiotroph	NA	Hydrophobic soil	F, B	Spain, Botanical Garden	Zotti et al. 2021
*Floccularialuteovirens*	T2	NA	Fungal Chemicals	NA	P	China grassland	Cao et al. 2021
*Leucocalocybemongolica*	T2	Increase in microbial diversity.	Carbohydrates	NA	P, F, B	China grassland	Duan et al. 2021 2022
*Agaricusbisporus*	T2	NA	Amino acid accumulation, increased metabolism	NA	P	China grassland	Liu et al. 2021
*Tricholomamatsutake*	T0	Decreased microbial diversity.	NA	NA	F, B	South Korea, woodland	An et al. 2021
Multispecies	T2	Increase Gram+	K depletion	NA	F, B	Spain, grassland	Rodriguez et al. 2022
*Leucocalocybemongolica*	T2	Decreased microbial diversity.	Microbiome	NA	P, F, B	China grassland	Wang et al. 2022a b
NA	T1	Changes in microbiome	Nutrients	Fungal pathogen	P, F,B	China grassland	Li et al. 2022
*Lepistaluscina*	T2	Decreased fungal diversity	NA	NA	P, F	Mongolia, grassland	Xu et al. 2023
Multispecies	T2	Copiotrophs	Increase of C-degradation genes	NA	F, B	China grassland	Lui et al. 2023

Shantz and Piemeisel (1917) proposed that ammonium enrichment in FFR soil is transferred to plants in absorbable forms, which is thought to result from the decomposition of organic matter by the fungi. However, it has also been observed that slight ammonium enrichment occurs behind the mycelial mats, suggesting that the decomposition of senescent mycelium might contribute to this enrichment (Shantz and Piemeisel 1917; Bonanomi et al. 2012; Yang et al. 2019). Consequently, plants located near or behind the mycelial mats, if not affected by drought or cyanides, often show notable growth, with clear signs of soil fertilisation, such as higher leaf protein content (Albrecht et al. 1951; Rogers and McAllister 1969; Stelfox and Stelfox 1979) and shorter root lengths (Cao et al. 2021).

While the nutrient-based hypothesis remains plausible, there is limited evidence regarding the exact dynamics of nutrient adsorption by plants in FFRs. In *Agaricusgennadii* (Chatin & Boud.) P.D. Orton, for example, plant biomass was found to follow nutrient pools in the soil, with stimulation occurring under optimal N:P ratios (Yang et al. 2018b). However, in *M.oreades*, it was suggested that vegetation stimulation might be linked to elevated levels of nitrates, with roots absorbing nutrients through lateral extensions or through diffusion of nitrates from adjacent zones (Gramss et al. 2005). This hypothesis was not fully accepted, as high levels of carbonification may impair the ability of plants to absorb nitrogen compounds and nitrate diffusion was insufficient to explain the extent of the greener belts.

In *A.arvensis*, phosphorus and potassium enrichment in the soil was thought to support the formation of greener belts, but plants exhibited symptoms of nutrient deficiencies, particularly phosphorus (Edwards 1984; Edwards 1988). This led to the idea that these nutrients may be immobilised within the mycelium of the fungus rather than being transferred to the plants (Fisher 1977; Gramss et al. 2005).

Several studies have suggested additional processes beyond nutrient enrichment that contribute to the formation of greener belts in FFRs. One such process is the creation of a favourable microbiome that supports plant growth in the stimulated areas, which has been partially confirmed by the presence of beneficial microorganisms, such as *Trichoderma* Pers., *Burkholderia* Yabuuchi et al. and arbuscular mycorrhizal fungi (Zotti et al. 2020). Another contributing factor is the production of plant growth-promoting substances, referred to as “Fairy chemicals” (Mitchinson 2014). These chemicals, primarily isolated from the pure culture of *L.sordida*, include 2-azahypoxanthine (AHX), which promotes root and shoot elongation in plants by triggering up-regulation of genes involved in nutrient uptake, stress resistance, detoxification and pathogen resistance (Choi et al. 2010a; Choi et al. 2010b; Mitchinson 2014). These compounds, along with imidazole-4-carboxamide (ICA), have been shown to enhance plant growth and productivity under certain conditions (Choi et al. 2017; Takano et al. 2019). These molecules belong to an unknown family of plant hormones producing the plant growth promoter 2-aza-8-oxohypoxanthin (AOH). The bio-stimulants fairy chemicals were produced massively by synthesis, but with scarce yields (Ito et al. 2020). Instead, bioconversion of AHX in AOH by the resting cell of *Burkholderiacontaminans* Vanlaere et al. had high efficiency with a yield of 100% of transformation (Choi et al. 2016). More detailed knowledge on the topic of fairy chemicals is reviewed in Kawagishi (2019) and, more recently, in Liu et al. (2024).

Supporting the phytostimulant hypothesis, simulation studies have indicated that FFRs characterised by a stimulated vegetation belt are likely to be the result of volatile phytostimulants released into the soil (Table 1). These simulations suggest that, in addition to nutrient release, phytostimulation plays an important role in promoting plant growth in these areas (Salvatori et al. 2023).

### ﻿Effects on soil microbiota

The understanding of the effect of FFRs fungi on soil microbiota has evolved alongside advancements in microbiological techniques, reflecting the growing interest in this topic within modern research (Table 1). Early studies focused on the microbial characteristics associated with the formation of the greener belt of vegetation. Meanwhile, the soil microbial community in woodland ecosystems has been investigated since the isolation of mycorrhizal-helper bacteria in the *Shiro* of *T.matsutake*, representing useful information for the stable cultivation of valuable edible fungi and for reforestation (Powell 1993; Yun and Hall 2004).

Initially, the plate dilution method was the primary technique used to study the changes in soil microbiota induced by FFR fungi (Ohara and Hamada 1967; Norstadt et al. 1973). This method was later replaced by more advanced community-based techniques, including FAME profiling (Espeland et al. 2013), PCR-DGGE (Kataoka et al. 2012) and Next-Generation Sequencing (Marí et al. 2020; Duan and Bau 2021; Zotti et al. 2021).

Amongst culturable bacteria, the development of FFR mycelial mats is associated with a general simplification of the bacterial community in *T.matsutake* (Ohara and Hamada 1967; Kataoka et al. 2012). In contrast, in *M.oreades*, no significant changes are observed, although a reduction in enzymatic activities has been noted (Norstadt et al. 1973). Studies have demonstrated that FFR soils can harbour a large number of prokaryotic colonies (Gramss et al. 2005) and associated enzymes (Bonanomi et al. 2012). Using FAME profiling combined with DNA sequencing, it was found that, in soils colonised by the mycelial mats of *A.lilaceps*, *Pseudomonasfluorescens* Migula, *Stenotrophomonasmaltophilia* (Hugh) Palleroni & Bradbury and *Agrobacteriumradiobacter* (Beijerinck & van Delden) Conn were the most prominent species. However, there is limited information on the changes in fungal communities associated with culturable species studies, with the only exception being an increase in *Mortierella* Coem. in the soil of *T.matsutake* (Ohara and Hamada 1967), which highlights the limited affinity of many fungi for culture-based methodologies.

Field surveys combined with PCR technologies applied to woodland FFRs revealed that, during the passage of the fungal front, root tips are dominated by *T.matsutake*, suggesting competitive exclusion amongst fungal symbionts (Lian et al. 2006). Following this, the application of 454 pyrosequencing in soil colonised by *T.matsutake* revealed a strong simplification in the fungal community, except for the equitability amongst different taxa, which appeared unaffected (Kim et al. 2013). In contrast to the fungal community, bacterial communities appeared more responsive to the progression of the fungus, although no remarkable changes in diversity metrics were observed (Kim et al. 2014).

Contrary to these pioneering works, other studies from the same ecoregion reported differing results, indicating changes in both eukaryotic and prokaryotic microbial communities following the development of *T.matsutake*. No apparent changes were observed in the eukaryotic community, but specific changes were detected in the bacterial community in soils dominated by *T.matsutake*. Using various metrics, such as Bray-Curtis similarity for fungal communities and UniFrac distance for bacterial communities, the study found that geographic location was a better predictor of fungal community composition than the passage of the FFR fungus. In contrast, bacterial community composition showed a stronger association with the developmental stage of *T.matsutake*. Furthermore, some bacterial genera, such as *Burkholderia*, *Bacillus* Cohn and *Paenibacillus* Ash et al., exhibited a common advantageous response to the fungal front (Oh et al. 2016). These genera were subsequently evaluated as mycorrhizal helper bacteria under controlled conditions, revealing that most bacterial taxa, including *Burkholderia*, had negative effects on *T.matsutake* growth in Petri dishes, while species from the *Paenibacillus* and *Staphylococcus* Rosenbach genera promoted fungal growth (Oh and Lim 2018).

In Tibetan grasslands, the effects of *A.gennadii* and *A.campestris* on the bacterial community were evaluated across alpine and temperate climates within stimulated vegetation (Yang et al. 2018a, 2018b). The results indicated varying effects of FFR development on bacterial diversity, with one area showing increased diversity indices and another showing a decrease, though no specific bacterial associations were identified.

More recent studies have focused on the community structure associated with the development of *A.arvensis*. These studies revealed few significant changes in the number of OTUs, Shannon diversity index and Pilou’s evenness for the bacterial community (Zotti et al. 2020). However, marked changes were observed in the fungal community, particularly in alpha diversity decreasing in fungal dominated soil. In the same study, a significant increase in *Burkholderia* amongst bacteria and *Trichoderma* amongst fungi is thought to result from a phenomenon of mycoparasitism. Evidence from other studies supports this idea, suggesting that *Burkholderia* can convert harmful toxins produced by the fungus into less toxic forms (Choi et al. 2016) and *Trichoderma* species are less sensitive to toxins produced by other fungal mycelium (Blenis et al. 2004). Both taxa also exhibit inhibitory effects on the growth of the associated fungal species (Oh and Lim 2018; Oh et al. 2018) and they are known for their role in biological control of fungal populations in agricultural fields (Kubicek et al. 2001; Parke and Gurian-Sherman 2001; Benítez et al. 2004). These findings suggest that the persistence of these taxa in FFRs may represent an evolutionary advantage developed to counteract the defensive strategies of dominant fungal mycelium. However, direct evidence of mycoparasitism is still required to fully understand the role of *Burkholderia* in FFRs.

Further studies emphasise the impact of FFR fungi on grassland ecosystems, particularly in terms of increasing microbial species richness. A study on FFR-forming fungi in Spanish grasslands revealed that, within the rings, relative abundances of *Pleosporales* Luttr. ex M.E. Barr and *Eurotiales* G.W. Martin ex Benny & Kimbr. decreased, while Clavaria Vaill. ex L., *Psathyrella* (Fr.) Quél., *Tricholoma* (Fr.) Staude, *Amanita* Pers. and *Lycoperdon* Pers. genera increased (Marí et al. 2020). The authors suggested that fungal diversity increases within the rings, although the interpretation of results requires caution, as ectomycorrhizal species such as *Tricholoma* and *Amanita* could contribute to the genetic signal detected, possibly from spores in the soil, as observed for *Cantharellus* Adans. ex Fr. in the inner zone of *A.arvensis*FFRs (Zotti et al. 2020).

In FFRs of *Leucocalocybemongolica* (S. Imai) Z.M. He & Zhu L. Yang in the Mongolian Tibetan Plateau, higher species richness was observed in the zone of stimulated vegetation compared to the surrounding grassland. This increase in richness was more pronounced in the bacterial community than the fungal community (Duan and Bau 2021). Similar increases in bacterial species richness in areas with active mycelial mats have been reported in other metagenomic-based studies on FFRs (Zotti et al. 2021). In FFRs of *C.gambosa*, an association between the bacteria belonging to genera such as *Pseudomonas* Migula, *Sphingobacterium* Yabuuchi, *Pedobacter* Steyn, *Parapedobacter* Kim, *Advenella* Coenye and *Rhodanobacter* Nalin were observed. The authors, integrating metagenomic data with soil physiochemical data, hypothesised that the high levels of ammonia and nitrate around the mycelial mats could be the result of consortia with soil bacteria, rather than being solely produced by fungal saprobic activity. This supports the idea that dominant fungal fronts should be considered holobionts, formed not just by a single organism, but by a complex consortium of microbial taxa (Zotti et al. 2021).

## ﻿Concluding remarks

Fungi play essential roles in ecosystem functioning, directly contributing to biogeochemical cycles and performing critical functions in the structuring of plant communities. They act as symbionts, pathogens or saprotrophs, influencing plant growth and decay (Paul 2014; Dighton 2016; Tedersoo et al. 2020). Due to their cryptic nature, fungi’s actions in terrestrial environments are often difficult to observe. This is particularly true for *Basidiomycetes*, which can either facilitate the formation of monospecific plant communities through ectomycorrhizal symbiosis (Bennett et al. 2017; Corrales et al. 2018) or, when pathogenic, contribute to the formation of decaying vegetation zones (Anselmi and Minerbi 1988; Legrand et al. 1996; Ferguson et al. 2003; Bendel et al. 2006).

The study of FFRs provides a valuable opportunity to delve deeper into the complex field of soil and fungal ecology, bridging multiple scientific disciplines such as mycology, microbiology, chemistry and botany. Norstadt (1973) outlined several advantages of studying FFRs, which include:

1. each FFR serving as a replicate within a specific species of grass and soil type;
2. observable changes in soil properties within short distances;
3. the long lifespan of the fungus;
4. seasonal fluctuations in fungal activity;
5. vegetation responses and soil effects that help locate the fungus;
6. the possibility of transferring sod sections to greenhouses or growth chambers for further study;
7. the potential to isolate and cultivate the fungus in pure culture.

In some cases, visualising these patterns under field conditions provides invaluable insight into fungal population dynamics that would otherwise be challenging to study (Abesha et al. 2003). However, the role of FFRs in modulating species richness in ecosystems remains underexplored and warrants focused research. Further investigations into fungal species specificity and the influence of environmental conditions are needed (Carney and Matson 2005). Additionally, the role of volatile compounds in FFR dynamics deserves attention. For example, *C.gambosa* produces an intense floury odour, while other fungi such as *M.oreades*, *I.geotropa* and *A.arvensis* release cyanide/anise-smelling compounds. Fungi like *T.matsutake*, *C.nebularis* and *Agaricuscrocodilinus* Murrill produce complex, intense and hard-to-define odours. As FFR-forming fungi, it would be intriguing to determine whether their volatilome contributes to the formation of dominant fungal fronts and plays a role in inhibiting or stimulating associated plant communities.

Seasonal dynamics and changes in the soil microbiota and plant communities in relation to the configuration of mycelial mats are still insufficiently studied. It can be argued that turf management practices, such as irrigation, may influence the position of mycelial mats by optimising moisture conditions or avoiding hypoxic zones due to stagnant water (Griffin 1963; Miller et al. 1989). Such studies could provide valuable insights into whether mycelial mats can migrate across soil horizons.

Another area of limited research is the molecular mechanisms through which FFR fungi exert their effects. The use of next-generation sequencing technologies, such as shotgun sequencing, offers a promising avenue for unravelling how FFR fungi act as ecosystem engineers, regulating species co-existence at both the soil microbiome and plant community levels.

**Figure 9.**
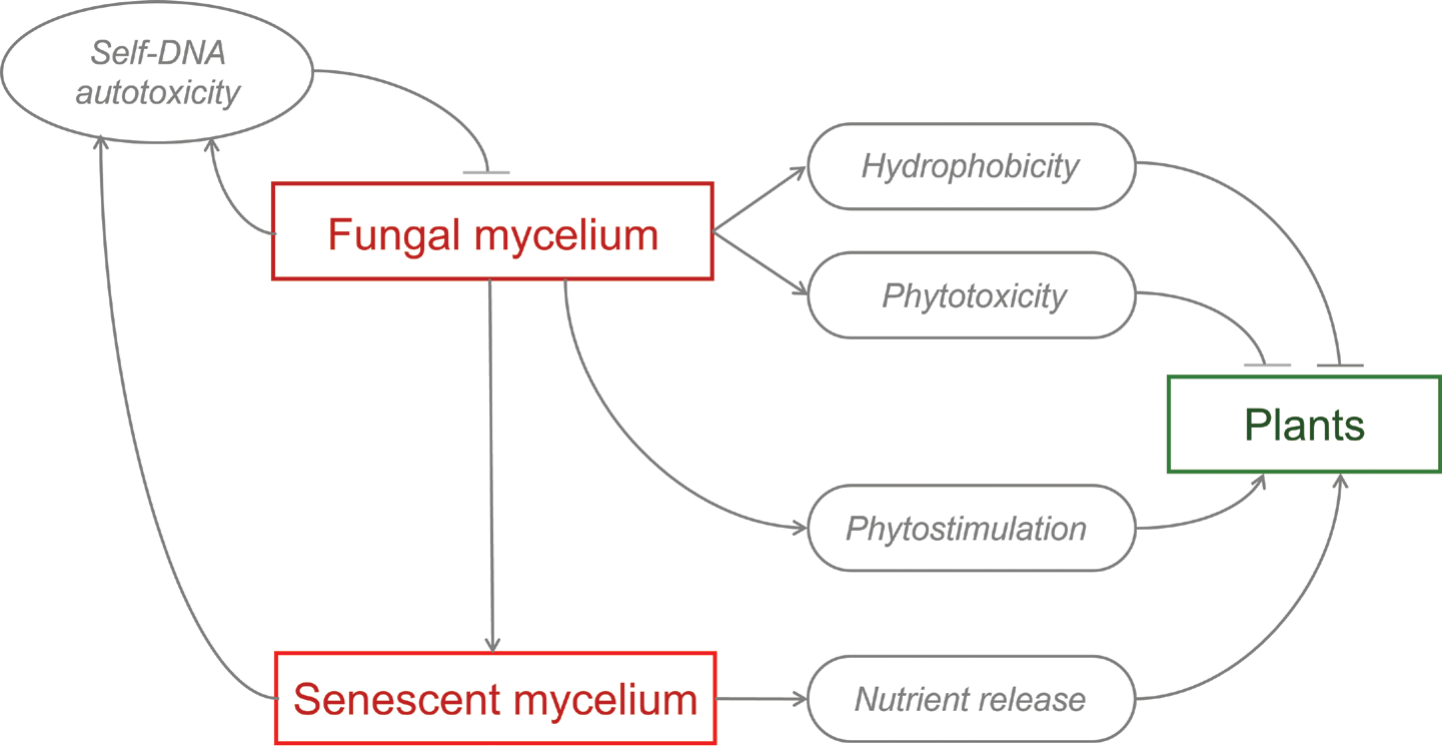
Schematic model diagram of the main fungal fairy rings functional processes (modified from Salvatori et al. 2023). Arrows indicate positive relationships. Truncated connectors show negative effects. The spatial position and intensity of each process determines the development of the different pattern types.

The recent study by Salvatori et al. (2023) has provided important insights. It suggests that the development of different ring patterns results from the co-occurrence of two distinct processes (Fig. 9). The first involves the fungal front ring, which is associated with autotoxicity caused by self-DNA accumulation, a phenomenon observed in plants (Bonanomi et al. 2014; Cartenì et al. 2016). The second process is related to the effects of the fungus on vegetation, which can be attributed to the direct action of the fungus such as the release of phytotoxic compounds and the induction of hydrophobicity in the soil by the mycelial mats or to nutrient flush released by decomposing mycelium after the onset of its self-inhibition.

However, the deep understanding of the developmental mechanisms has perhaps cleared the explanation of fairy rings formation, but such removal of thin magic halo has certainly not reduced the wonder for the beauty of nature in its ever surprisingly dynamic pattern and intertwined complex systems.

## ﻿Additional information

### Adherence to national and international regulations

No Fungal strains were used in this study.

### Author ORCIDs

Maurizio Zotti https://orcid.org/0000-0001-5540-9477

Giuliano Bonanomi https://orcid.org/0000-0002-1831-4361

Stefano Mazzoleni https://orcid.org/0000-0002-1132-2625

### Data availability

All of the data that support the findings of this study are available in the main text or Supplementary Information.

## Supplementary materials

Supplementary material 1 List of records on FFR-forming fungi for different taxonomic levels and ecological functions available in literature Data type pdf https://binary.pensoft.net/file/1253387This dataset is made available under the Open Database License (http://opendatacommons.org/licenses/odbl/1.0/). The Open Database License (ODbL) is a license agreement intended to allow users to freely share, modify, and use this Dataset while maintaining this same freedom for others, provided that the original source and author(s) are credited.Maurizio Zotti
