# Supplementary material for: ﻿Fungal fairy rings: history, ecology, dynamics and engineering functions
Source: IMA Fungus. 2025 Feb 17;16:e138320. doi: 10.3897/imafungus.16.138320 (PMC11881004; doi:10.3897/imafungus.16.138320)
Supplement: Supplementary material 1 — List of records on FFR-forming fungi for different taxonomic levels and ecological functions available in literature [file imafungus-16-e138320-s001.pdf]

List of records on FFR-forming fungi for different taxonomic levels and ecological functions available in literature.

Phylum	Genus	Name reported	Current name	ecology	reference	year
Basidiomycota	*Marasmius*	*Marasmius oreades*	*Marasmius oreades*	Sph	Abesha, (2003)	2003
Basidiomycota	*Calocybe*	*Calocybe gambosa*	*Calocybe gambosa*	Sph	Ainsworth and Bysby, (1950)	1950
Basidiomycota	*Marasmius*	*Marasmius oreades*	*Marasmius oreades*	Sph	Albrecht, (1951)	1951
Basidiomycota	*Marasmius*	*Marasmius oreades*	*Marasmius oreades*	Sph	Ayer (1989)	1989
Basidiomycota	*Infundibulocybe*	*Leucopaxillus geotropa*	*Infundibulocybe geotropa*	Sph	Becker, (1953)	1953
Basidiomycota	*Marasmius*	*Marasmius oreades*	*Marasmius oreades*	Sph	Blenis, (1997)	1997
Basidiomycota	*Marasmius*	*Marasmius oreades*	*Marasmius oreades*	Sph	Blenis, (2004)	2004
Basidiomycota	*Marasmius*	*Marasmius oreades*	*Marasmius oreades*	Sph	Blenis, (2004)	2004
Basidiomycota	*Agaricus*	*Agaricus campestris*	*Agaricus campestris*	Sph	Bonanomi, (2012)	2012
Basidiomycota	*Marasmius*	*Marasmius oreades*	*Marasmius oreades*	Sph	Burnett and Evans, (1966)	1966
Basidiomycota	*Marasmius*	*Marasmius oreades*	*Marasmius oreades*	Sph	Caspar and Spitteler (2015)	2015
Basidiomycota	*Floccularia*	*Floccularia luteovirens*	*Floccularia luteovirens*	Sph	Chen, (2000)	2000
Basidiomycota	*Lepista*	*Lepista sordida*	*Lepista sordida*	Sph	Choi, (2010a)	2010
Basidiomycota	*Lepista*	*Lepista sordida*	*Lepista sordida*	Sph	Choi, (2010b)	2010
Basidiomycota	*Marasmius*	*Marasmius oreades*	*Marasmius oreades*	Sph	Cosby, (1959)	1959
Basidiomycota	*Leucocalocybe*	*Tricholoma monogolicum*	*Leucocalocybe mongolica*	sph	Dhiao, (2004)	2004
Basidiomycota	*Marasmius*	*Marasmius oreades*	*Marasmius oreades*	Sph	Dickinson, (1979)	1979
Basidiomycota	*Clitocybe*	*Clitocibe nebularis*	*Clitocybe nebularis*	Sph	Dowson (1989)	1989
Basidiomycota	*Leucocalocybe*	*Leucocalocybe mongolica*	*Leucocalocybe mongolica*	Sph	Duan, (2021a)	2021
Basidiomycota	*Leucocalocybe*	*Leucocalocybe mongolica*	*Leucocalocybe mongolica*	Sph	Duan, (2021b)	2021
Basidiomycota	*Agaricus*	*Agaricus arvensis*	*Agaricus arvensis*	Sph	Edwards, (1984)	1984
Basidiomycota	*Agaricus*	*Agaricus arvensis*	*Agaricus arvensis*	Sph	Edwards, (1988)	1988
Basidiomycota	*Lycoperdon*	*Lycoperdon sp.*	*Lycoperdon sp.*	Sph	Elliot, (2002)	2002
Basidiomycota	*Agaricus*	*Agaricus lilaceps*	*Agaricus lilaceps*	Sph	Espeland, (2013)	2013
Basidiomycota	*Gymnopus*	*Marasmius dryophilus*	*Gymnopus dryophilus*	Sph	Falandysz, (2013)	2013
Basidiomycota	*Gymnopus*	*Gymnopus erythropus*	*Gymnopus erythropus*	Sph	Falandysz, (2013)	2013
Basidiomycota	*Marasmius*	*Marasmius oreades*	*Marasmius oreades*	Sph	Fidanza, (2007)	2007
Basidiomycota	*Marasmius*	*Marasmius oreades*	*Marasmius oreades*	Sph	Filer, (1965a)	1965
Basidiomycota	*Marasmius*	*Marasmius oreades*	*Marasmius oreades*	Sph	Filer, (1965b)	1965
Basidiomycota	*Marasmius*	*Marasmius oreades*	*Marasmius oreades*	Sph	Fisher, (1976)	1976
Basidiomycota	*Marasmius*	*Marasmius oreades*	*Marasmius oreades*	Sph	Fox, (2006)	2006
Basidiomycota	*Mycena*	*Mycena galopus*	*Mycena galopus*	sph	Frankland, (1998)	1998
Basidiomycota	*Marasmius*	*Marasmius oreades*	*Marasmius oreades*	Sph	Gramss, (2005)	2005
Basidiomycota	*Calocybe*	*Calocybe camgosa*	*Calocybe gambosa*	Sph	Guminska, (1976)	1976
Basidiomycota	*Clitocybe*	*Clitocybe dealbata*	*Clitocybe rivulosa*	Sph	Guminska, (1976)	1976
Basidiomycota	*Cystoderma*	*Cystoderma amianthinum*	*Cystoderma amianthinum*	Sph	Guminska, (1976)	1976
Basidiomycota	*Lactarius*	*Lactarius semisanguifluus*	*Lactarius semisanguifluus*	Sym	Guminska, (1976)	1976
Basidiomycota	*Lepista*	*Lepista nuda*	*Lepista nuda*	Sph	Guminska, (1976)	1976
Basidiomycota	*Macrolepiota*	*Macrolepiota mastoidea*	*Macrolepiota mastoidea*	Sph	Guminska, (1976)	1976
Basidiomycota	*Macrolepiota*	*Macrolepiota procera*	*Macrolepiota procera*	Sph	Guminska, (1976)	1976
Basidiomycota	*Marasmius*	*Marasmius oreades*	*Marasmius oreades*	Sph	Guminska, (1976)	1976

Basidiomycota	*Pseudoclitocybe*	*Clitocybe expallens*	*Pseudoclitocybe expallens*	Sph	Guminska, (1976)	1976
Basidiomycota	*Agaricus*	*Agaricus arvensis*	*Agaricus arvensis*	Sph	Halinsky and Peterson, (1970)	1970
Basidiomycota	*Agaricus*	*Agaricus campestris*	*Agaricus campestris*	Sph	Halinsky and Peterson, (1970)	1970
Basidiomycota	*Calvatia*	*Calvatia cyathiformis*	*Calvatia cyathiformis*	Sph	Halinsky and Peterson, (1970)	1970
Basidiomycota	*Clitocybe*	*Clitocybe caespitosa*	*Clitocybe caespitosa*	Sph	Halinsky and Peterson, (1970)	1970
Basidiomycota	*Clitocybe*	*Clitocybe dealbata*	*Clitocybe rivulosa*	Sph	Halinsky and Peterson, (1970)	1970
Basidiomycota	*Leucoagaricus*	*Lepiota naucina*	*Leucoagaricus leucolithes*	Sph	Halinsky and Peterson, (1970)	1970
Basidiomycota	*Leucopaxillus*	*Clitocibe gigantea*	*Leucopaxillus giganteus*	Sph	Halinsky and Peterson, (1970)	1970
Basidiomycota	*Marasmius*	*Marasmius oreades*	*Marasmius oreades*	Sph	Halinsky and Peterson, (1970)	1970
Basidiomycota	*Panaeolina*	*Psilocybe foenisecii*	*Panaeolina foenisecii*	Sph	Halinsky and Peterson, (1970)	1970
Basidiomycota	*Panaeolus*	*Panaeolus retirugis*	*Panaeolus papilionaceus*	Sph	Halinsky and Peterson, (1970)	1970
Basidiomycota	*Marasmius*	*Marasmius oreades*	*Marasmius oreades*	Sph	Hardwick and Heard, (1978)	1978
Basidiomycota	*Albatrellopsis*	*Polyporus confluens*	*Albatrellopsis confluens*	Sym	Hawksworth, (1962)	1962
Basidiomycota	*Clitocybe*	*Clitocybe nebularis*	*Clitocybe nebularis*	Sph	Hearst, (2013)	2013
Basidiomycota	*Marasmius*	*Marasmius oreades*	*Marasmius oreades*	sph	Hiltunen, (2019)	2019
Basidiomycota	*Lepista*	*Lepista nuda*	*Lepista nuda*	Sph	Hjelm, (1994)	1994
Basidiomycota	*Marasmius*	*Marasmius oreades*	*Marasmius oreades*	Sph	Ingold, (1974)	1974
Basidiomycota	*Floccularia*	*Armillaria luteovirens*	*Floccularia luteovirens*	Sph	Jinyang, (2005)	2005
Basidiomycota	*Tricholoma*	*Tricholoma matzutake*	*Tricholoma nauseosmus*	Sym	Katooka, (2012)	2012
Basidiomycota	*Tricholoma*	*Tricholoma matzutake*	*Tricholoma nauseosmus*	Sym	Kim, (2013)	2013
Basidiomycota	*Tricholoma*	*Tricholoma matzutake*	*Tricholoma nauseosmus*	Sym	Kim, (2014)	2014
Basidiomycota	*Tricholoma*	*Tricholoma matzutake*	*Tricholoma nauseosmus*	Sym	Koo, (2009)	2009
Basidiomycota	*Clitocybe*	*Clitocybe nebularis*	*Clitocybe nebularis*	sph	Kosanic, (2020)	2020
Basidiomycota	*Infundibulocybe*	*Clitocybe geotropa*	*Infundibulocybe geotropa*	sph	Kosanic, (2020)	2020
Basidiomycota	*Hygrophorus*	*Hygrophorus agathosmus*	*Hygrophorus agathosmus*	Sph	Larsson, (2018)	2018
Basidiomycota	*Marasmius*	*Marasmius oreades*	*Marasmius oreades*	Sph	Lebeau, 1961	1961
Basidiomycota	*Marasmius*	*Marasmius oreades*	*Marasmius oreades*	Sph	Lebeau, 1963	1963
Basidiomycota	*Marasmius*	*Marasmius oreades*	*Marasmius oreades*	Sph	Lebeau, 1963b	1963
Basidiomycota	*Tricholoma*	*Tricholoma matzutake*	*Tricholoma nauseosmus*	Sym	Li, (2016)	2016
Basidiomycota	*Tricholoma*	*Tricholoma matzutake*	*Tricholoma nauseosmus*	Sym	Lian, (2006)	2006
Basidiomycota	*Agaricus*	*Agaricus bisporus*	*Agaricus bisporus*	Sph	Lui, (2021)	2021
Basidiomycota	*Marasmius*	*Marasmius oreades*	*Marasmius oreades*	Sph	Mallet, (1988)	1988
Basidiomycota	*Marasmius*	*Marasmius oreades*	*Marasmius oreades*	Sph	Mathur, (1970)	1970
Basidiomycota	*Marasmius*	*Marasmius oreades*	*Marasmius oreades*	Sph	Miles, (1971)	1971
Basidiomycota	*Agaricus*	*Agaricus argenteus*	*Agaricus argenteus*	Sph	Miller and Gongloff, (2021)	2021
Basidiomycota	*Agaricus*	*Agaricus lilaceps*	*Agaricus lilaceps*	Sph	Miller and Gongloff, (2021)	2021
Basidiomycota	*Bovista*	*Bovista plumbea*	*Bovista plumbea*	Sph	Miller and Gongloff, (2021)	2021
Basidiomycota	*Calvatia*	*Calvatia chilensis*	*Calvatia chilensis*	Sph	Miller and Gongloff, (2021)	2021
Basidiomycota	*Calvatia*	*Calvatia fragilis*	*Calvatia cyathiformis*	Sph	Miller and Gongloff, (2021)	2021
Basidiomycota	*Disciseda*	*Disciseda candida*	*Disciseda candida*	Sph	Miller and Gongloff, (2021)	2021

Basidiomycota	*Geastrum*	*Geastrum sp.*	*Geastrum sp.*	Sph	Miller and Gongloff, (2021)	2021
Basidiomycota	*Lepiota*	*Lepiota erminea*	*Lepiota erminea*	Sph	Miller and Gongloff, (2021)	2021
Basidiomycota	*Leucocalocybe*	*Leucocalocybe mongolica*	*Leucocalocybe mongolica*	Sph	Miller and Gongloff, (2021)	2021
Basidiomycota	*Lycoperdon*	*Lycoperdon dermoxanthum*	*Lycoperdon dermoxanthum*	Sph	Miller and Gongloff, (2021)	2021
Basidiomycota	*Marasmius*	*Marasmius oreades*	*Marasmius oreades*	Sph	Miller and Gongloff, (2021)	2021
Basidiomycota	*Tulostoma*	*Tulostoma calcareum*	*Tulostoma calcareum*	Sph	Miller and Gongloff, (2021)	2021
Basidiomycota	*Agaricus*	*Agaricus arvensis*	*Agaricus arvensis*	Sph	Miller, (2012)	2012
Basidiomycota	*Agaricus*	*Agaricus campestris*	*Agaricus campestris*	Sph	Miller, (2012)	2012
Basidiomycota	*Arachnion*	*Arachnion album*	*Arachnion album*	Sph	Miller, (2012)	2012
Basidiomycota	*Coprinopsis*	*Coprinus kubickae*	*Coprinopsis kubickae*	Sph	Miller, (2012)	2012
Basidiomycota	*Coprinus*	*Coprinus comatus*	*Coprinus comatus*	Sph	Miller, (2012)	2012
Basidiomycota	*Coprinus*	*Coprinus sp*	*Coprinus sp*	Sph	Miller, (2012)	2012
Basidiomycota	*Cyclocybe*	*Agrocybe aegerita*	*Cyclocybe cilindracea*	Sph	Miller, (2012)	2012
Basidiomycota	*Lepista*	*Lepista nuda*	*Lepista nuda*	Sph	Miller, (2012)	2012
Basidiomycota	*Lepista*	*Lepista sordida*	*Lepista sordida*	Sph	Miller, (2012)	2012
Basidiomycota	*Lycoperdon*	*Vascellum curtisii*	*Lycoperdon curtisii*	Sph	Miller, (2012)	2012
Basidiomycota	*Lycoperdon*	*Bovista dermoxantha*	*Lycoperdon dermoxanthum*	Sph	Miller, (2012)	2012
Basidiomycota	*Lycoperdon*	*Bovista dermoxantha*	*Lycoperdon dermoxanthum*	Sph	Miller, (2012)	2012
Basidiomycota	*Lycoperdon*	*Lycoperdon marginatum*	*Lycoperdon marginatum*	Sph	Miller, (2012)	2012
Basidiomycota	*Marasmius*	*Marasmius graminum*	*Marasmius graminum*	Sph	Miller, (2012)	2012
Basidiomycota	*Marasmius*	*Marasmius oreades*	*Marasmius oreades*	Sph	Miller, (2012)	2012
Basidiomycota	*Marasmius*	*Marasmius siccus*	*Marasmius siccus*	Sph	Miller, (2012)	2012
Basidiomycota	*Marasmius*	*Marasmius sp.*	*Marasmius sp.*	Sph	Miller, (2012)	2012
Basidiomycota	*Parasola*	*Parasola pliicatilis*	*Parasola pliicatilis*	Sph	Miller, (2012)	2012
Basidiomycota	*Collybia*	*Collybia pinastris*	*Collybia pinastris*	SPh	Miyamoto, (2004)	2004
Basidiomycota	*Tricholoma*	*Tricholoma matzutake*	*Tricholoma nauseosmus*	Sym	Narimatsu, (2015)	2015
Basidiomycota	*Marasmius*	*Marasmius oreades*	*Marasmius oreades*	Sph	Northstat, (1973)	1973
Basidiomycota	*Tricholoma*	*Tricholoma matzutake*	*Tricholoma nauseosmus*	Sym	Oh, (2016)	2016
Basidiomycota	*Tricholoma*	*Tricholoma matzutake*	*Tricholoma nauseosmus*	Sym	Oh, (2018)	2018
Basidiomycota	*Tricholoma*	*Tricholoma matzutake*	*Tricholoma nauseosmus*	Sym	Ohara, (1967)	1967
Basidiomycota	*Coprinopsis*	*Coprinus kubickae*	*Coprinopsis kubickae*	Sph	RedHead, (1981)	1981
Basidiomycota	*Agaricus*	*Agaricus arvensis*	*Agaricus arvensis*	Sph	Rogers, (1969)	1969
Basidiomycota	*Agaricus*	*Agaricus campestris*	*Agaricus campestris*	Sph	Shantz and Piezemel, (1917)	1917
Basidiomycota	*Agaricus*	*Agaricus tabularis*	*Agaricus praerimosus*	Sph	Shantz and Piezemel, (1917)	1917
Basidiomycota	*Calvatia*	*Calvatia cyathiformis*	*Calvatia cyathiformis*	Sph	Shantz and Piezemel, (1917)	1917
Basidiomycota	*Calvatia*	*Calvatia fragilis*	*Calvatia cyathiformis*	Sph	Shantz and Piezemel, (1917)	1917
Basidiomycota	*Calvatia*	*Calvatia polygonia*	*Calvatia polygonia*	Sph	Shantz and Piezemel, (1917)	1917
Basidiomycota	*Chlorophyllum*	*Lepiota morganii*	*Chlorophyllum morganii*	Sph	Shantz and Piezemel, (1917)	1917
Basidiomycota	*Disciseda*	*Catastoma subterraneum*	*Disciseda subterranea*	Sph	Shantz and Piezemel, (1917)	1917
Basidiomycota	*Lycoperdon*	*Lycoperdon wrightii*	*Lycoperdon wrightii*	Sph	Shantz and Piezemel, (1917)	1917
Basidiomycota	*Melanoleuca*	*Tricholoma malaleuca*	*Melanoleuca malaleuca*	Sph	Shantz and Piezemel, (1917)	1917

Basidiomycota	*Agaricus*	*Psaliota arvensis*	*Agaricus arvensis*	Sph	Shantz and Piezemel, (1917)*	1862
Basidiomycota	*Agaricus*	*Psaliota arvensis*	*Agaricus arvensis*	Sph	Shantz and Piezemel, (1917)*	1869
Basidiomycota	*Agaricus*	*Agaricus arvensis*	*Agaricus arvensis*	Sph	Shantz and Piezemel, (1917)*	1900
Basidiomycota	*Agaricus*	*Agaricus arvensis*	*Agaricus arvensis*	Sph	Shantz and Piezemel, (1917)*	1906
Basidiomycota	*Agaricus*	*Agaricus campestris*	*Agaricus campestris*	Sph	Shantz and Piezemel, (1917)*	1807
Basidiomycota	*Agaricus*	*Agaricus campestris*	*Agaricus campestris*	Sph	Shantz and Piezemel, (1917)*	1884
Basidiomycota	*Agaricus*	*Agaricus campestris*	*Agaricus campestris*	Sph	Shantz and Piezemel, (1917)*	1901
Basidiomycota	*Agaricus*	*Agaricus campestris*	*Agaricus campestris*	Sph	Shantz and Piezemel, (1917)*	1910
Basidiomycota	*Agaricus*	*Agaricus sp.*	*Agaricus sp.*	Sph	Shantz and Piezemel, (1917)*	1917
Basidiomycota	*Amanita*	*Amanita muscaria*	*Amanita muscaria*	Sym	Shantz and Piezemel, (1917)*	1906
Basidiomycota	*Amanita*	*Amanita phalloides*	*Amanita phalloides*	Sym	Shantz and Piezemel, (1917)*	1906
Basidiomycota	*Bovista*	*Lycoperdon bovista*	*Bovista plumbea*	Sph	Shantz and Piezemel, (1917)*	1807
Basidiomycota	*Calocybe*	*Tricholoma gambosum*	*Calocybe gambosa*	Sph	Shantz and Piezemel, (1917)*	1847
Basidiomycota	*Cantharellus*	*Cantharellus cibarius*	*Cantharellus cibarius*	Sym	Shantz and Piezemel, (1917)*	1869
Basidiomycota	*Cantharellus*	*Cantharellus cinereus*	*Cantharellus cinereus*	Sym	Shantz and Piezemel, (1917)*	1906
Basidiomycota	*Chlorophyllum*	*Lepiota morganii*	*Chlorophyllum morganii*	Sph	Shantz and Piezemel, (1917)*	1887
Basidiomycota	*Chlorophyllum*	*Lepiota morganii*	*Chlorophyllum morganii*	Sph	Shantz and Piezemel, (1917)*	1917
Basidiomycota	*Clavaria*	*Clavaria sp.*	*Clavaria sp.*	Sph	Shantz and Piezemel, (1917)*	1914
Basidiomycota	*Clitocybe*	*Lepista nebularis*	*Clitocybe nebularis*	Sph	Shantz and Piezemel, (1917)*	1900
Basidiomycota	*Clitopilus*	*Agaricus prunulus*	*Clitopilus prunulus*	Sph	Shantz and Piezemel, (1917)*	1884
Basidiomycota	*Collybia*	*Collybia sp.*	*Collybia sp.*	Sph	Shantz and Piezemel, (1917)*	1914
Basidiomycota	*Collybiopsis*	*Collybia confluens*	*Collybiopsis confluens*	Sph	Shantz and Piezemel, (1917)*	1869
Basidiomycota	*Collybiopsis*	*Marasmius urens*	*Collybiopsis peronata*	Sph	Shantz and Piezemel, (1917)*	1869
Basidiomycota	*Cortinarius*	*Telamonia armillata*	*Cortinarius armillatus*	Sym	Shantz and Piezemel, (1917)*	1906
Basidiomycota	*Cortinarius*	*Inoloma traganum*	*Cortinarius traganus*	Sym	Shantz and Piezemel, (1917)*	1906
Basidiomycota	*Cuphophyllus*	*Hygrophorus virgineus*	*Cuphophyllus virgineus*	Sph	Shantz and Piezemel, (1917)*	1869
Basidiomycota	*Hebeloma*	*Agaricus crustuliniformis*	*Hebeloma crustuliniforme*	Sym	Shantz and Piezemel, (1917)*	1869
Basidiomycota	*Hydnellum*	*Hydnellum suaveolens*	*Hydnellum suaveolens*	Sym	Shantz and Piezemel, (1917)*	1905
Basidiomycota	*Hydnellum*	*Hydnellum suaveolens*	*Hydnellum suaveolens*	Sym	Shantz and Piezemel, (1917)*	1911
Basidiomycota	*Hydnum*	*Hydnum compactum*	*Hydnum compactum*	Sym	Shantz and Piezemel, (1917)*	1906
Basidiomycota	*Hydnum*	*Hydnum repandum*	*Hydnum repandum*	Sym	Shantz and Piezemel, (1917)*	1869
Basidiomycota	*Hydnum*	*Hydnum repandum*	*Hydnum repandum*	Sym	Shantz and Piezemel, (1917)*	1906
Basidiomycota	*Hygrophoropsis*	*Cantharellus aurantiacus*	*Hygrophoropsis aurantiaca*	Sph	Shantz and Piezemel, (1917)*	1906
Basidiomycota	*Infundibulicybe*	*Clitocybe infundibuliformis*	*Infundibulicybe gibba*	Sph	Shantz and Piezemel, (1917)*	1869
Basidiomycota	*Infundibulocybe*	*Agaricus geotropus*	*Infundibulocybe geotropa*	Sph	Shantz and Piezemel, (1917)*	1869
Basidiomycota	*Infundibulocybe*	*Agaricus maximus*	*Infundibulocybe geotropa*	Sph	Shantz and Piezemel, (1917)*	1914

Basidiomycota	*Inocybe*	*inocybe sp.*	*Inocybe sp.*	Sym	Shantz and Piezemel, (1917)*	1887
Basidiomycota	*Lactarius*	*Lactarius insulsus*	*Lactarius insulsus*	Sym	Shantz and Piezemel, (1917)*	1906
Basidiomycota	*Lactarius*	*Lactarius torminosus*	*Lactarius torminosus*	Sym	Shantz and Piezemel, (1917)*	1906
Basidiomycota	*Lactifluus*	*Lactifluus piperatus*	*Lactifluus piperatus*	Sym	Shantz and Piezemel, (1917)*	1869
Basidiomycota	*Lepista*	*Agaricus bicolor*	*Lepista nuda*	sph	Shantz and Piezemel, (1917)*	1869
Basidiomycota	*Lepista*	*Agaricus bicolor*	*Lepista nuda*	sph	Shantz and Piezemel, (1917)*	1870
Basidiomycota	*Lepista*	*Agaricus bicolor*	*Lepista nuda*	Sph	Shantz and Piezemel, (1917)*	1914
Basidiomycota	*Leucopaxillus*	*Clitocybe gigantea*	*Leucopaxillus giganteus*	Sph	Shantz and Piezemel, (1917)*	1862
Basidiomycota	*Leucopaxillus*	*Clitocybe gigantea*	*Leucopaxillus giganteus*	Sph	Shantz and Piezemel, (1917)*	1869
Basidiomycota	*Leucopaxillus*	*Clitocybe gigantea*	*Leucopaxillus giganteus*	Sph	Shantz and Piezemel, (1917)*	1884
Basidiomycota	*Leucopaxillus*	*Clitocybe gigantea*	*Leucopaxillus giganteus*	Sph	Shantz and Piezemel, (1917)*	1901
Basidiomycota	*Leucopaxillus*	*Clitocybe gigantea*	*Leucopaxillus giganteus*	Sph	Shantz and Piezemel, (1917)*	1911
Basidiomycota	*Lycoperdon*	*Lycoperdon cyclicum*	*Lycoperdon cyclicum*	Sph	Shantz and Piezemel, (1917)*	1898
Basidiomycota	*Lycoperdon*	*Lycoperdon gemmatum*	*Lycoperdon perlatum*	Sph	Shantz and Piezemel, (1917)*	1906
Basidiomycota	*Macrolepiota*	*Agaricus proceris*	*Macrolepiota procera*	Sph	Shantz and Piezemel, (1917)*	1807
Basidiomycota	*Marasmius*	*Marasmius oreades*	*Marasmius oreades*	Sph	Shantz and Piezemel, (1917)*	1796
Basidiomycota	*Marasmius*	*Marasmius oreades*	*Marasmius oreades*	Sph	Shantz and Piezemel, (1917)*	1807
Basidiomycota	*Marasmius*	*Marasmius oreades*	*Marasmius oreades*	Sph	Shantz and Piezemel, (1917)*	1862
Basidiomycota	*Marasmius*	*Marasmius oreades*	*Marasmius oreades*	Sph	Shantz and Piezemel, (1917)*	1869
Basidiomycota	*Marasmius*	*Marasmius oreades*	*Marasmius oreades*	Sph	Shantz and Piezemel, (1917)*	1870
Basidiomycota	*Marasmius*	*Marasmius oreades*	*Marasmius oreades*	Sph	Shantz and Piezemel, (1917)*	1883
Basidiomycota	*Marasmius*	*Marasmius oreadum*	*Marasmius oreades*	Sph	Shantz and Piezemel, (1917)*	1884
Basidiomycota	*Marasmius*	*Marasmius oreades*	*Marasmius oreades*	Sph	Shantz and Piezemel, (1917)*	1884
Basidiomycota	*Marasmius*	*Marasmius oreades*	*Marasmius oreades*	Sph	Shantz and Piezemel, (1917)*	1887
Basidiomycota	*Marasmius*	*Marasmius oreades*	*Marasmius oreades*	Sph	Shantz and Piezemel, (1917)*	1898
Basidiomycota	*Marasmius*	*Marasmius oreades*	*Marasmius oreades*	Sph	Shantz and Piezemel, (1917)*	1901
Basidiomycota	*Marasmius*	*Marasmius oreades*	*Marasmius oreades*	Sph	Shantz and Piezemel, (1917)*	1906
Basidiomycota	*Marasmius*	*Marasmius oreades*	*Marasmius oreades*	Sph	Shantz and Piezemel, (1917)*	1906
Basidiomycota	*Marasmius*	*Marasmius oreades*	*Marasmius oreades*	Sph	Shantz and Piezemel, (1917)*	1910
Basidiomycota	*Marasmius*	*Marasmius oreades*	*Marasmius oreades*	Sph	Shantz and Piezemel, (1917)*	1910
Basidiomycota	*Marasmius*	*Marasmius oreades*	*Marasmius oreades*	Sph	Shantz and Piezemel, (1917)*	1910
Basidiomycota	*Marasmius*	*Marasmius oreades*	*Marasmius oreades*	Sph	Shantz and Piezemel, (1917)*	1911
Basidiomycota	*Melanoleuca*	*Tricholoma grammopodium*	*Melanoleuca grammopodia*	Sph	Shantz and Piezemel, (1917)*	1869
Ascomycota	*Morchella*	*Morchella esculenta*	*Morchella esculenta*	Sph	Shantz and Piezemel, (1917)*	1906
Ascomycota	*Morchella*	*Morchella hybrida*	*Morchella semilibera*	Sph	Shantz and Piezemel, (1917)*	1906
Basidiomycota	*Paxillus*	*Paxillus involutus*	*Paxillus involutus*	Sph	Shantz and Piezemel, (1917)*	1906

Basidiomycota	*Pluteus*	*Pluteus cervinus*	*Pluteus cervinus*	Sph	Shantz and Piezemel, (1917)*	1917
Basidiomycota	*Suillus*	*Boletus bovinus*	*Suillus bovinus*	Sym	Shantz and Piezemel, (1917)*	1906
Basidiomycota	*Suillus*	*Boletus cavipes*	*Suillus cavipes*	Sym	Shantz and Piezemel, (1917)*	1906
Basidiomycota	*Suillus*	*Boletus elegans*	*Suillus elegnas*	Sym	Shantz and Piezemel, (1917)*	1906
Basidiomycota	*Suillus*	*Boletus variegatus*	*Suillus variegatus*	Sym	Shantz and Piezemel, (1917)*	1906
Basidiomycota	*Tricholoma*	*Tricholoma columbella*	*Tricholoma columbetta*	Sym	Shantz and Piezemel, (1917)*	1910
Basidiomycota	*Tricholoma*	*Tricholoma equestre*	*Tricholoma equestre*	Sym	Shantz and Piezemel, (1917)*	1917
Basidiomycota	*Tricholoma*	*Tricholoma sp.*	*Tricholoma sp.*	Sym	Shantz and Piezemel, (1917)*	1887
Basidiomycota	*Tricholoma*	*Agaricus terreus*	*Tricholoma terreum*	Sym	Shantz and Piezemel, (1917)*	1807
Ascomycota	*Tuber*	*Tuber sp.*	*Tuber sp.*	Sym	Shantz and Piezemel, (1917)*	1851
Basidiomycota	*Lycoperdon*	*Bovista dermoxantha*	*Lycoperdon dermoxanthum*	Sph	Terashima, (2004)	2004
Basidiomycota	*Lepista*	*Lepista sordida*	*Lepista sordida*	Sph	Terashima, (2007)	2007
Basidiomycota	*Agaricus*	*Agaricus campestris*	*Agaricus campestris*	Sph	Toohey, (1983)	1983
Basidiomycota	*Agaricus*	*Agaricus xanthoderma*	*Agaricus xanthodermus*	Sph	Toohey, (1983)	1983
Basidiomycota	*Amanita*	*Amanita flavorubescens*	*Amanita flavorubescens*	Sym	Toohey, (1983)	1983
Basidiomycota	*Amanita*	*Amanita muscaria*	*Amanita muscaria*	Sym	Toohey, (1983)	1983
Basidiomycota	*Calvatia*	*Calvatia fragilis*	*Calvatia cyathiformis*	Sph	Toohey, (1983)	1983
Basidiomycota	*Cantharellus*	*Cantharellus cibarius*	*Cantharellus cibarius*	Sym	Toohey, (1983)	1983
Basidiomycota	*Clavaria*	*Clavaria vermicularis*	*Clavaria fragilis*	Sph	Toohey, (1983)	1983
Basidiomycota	*Clavulina*	*Clavaria cinerea*	*Clavulina cinerea*	Sph	Toohey, (1983)	1983
Basidiomycota	*Clitocybe*	*Clitocybe dealbata*	*Clitocybe rivulosa*	sph	Toohey, (1983)	1983
Basidiomycota	*Cortinarius*	*Cortinarius sp*	*Cortinarius sp*	Sym	Toohey, (1983)	1983
Basidiomycota	*Cortinarius*	*Cortinarius sp 1*	*Cortinarius sp 1*	Sph	Toohey, (1983)	1983
Basidiomycota	*Cuphophyllus*	*Hygrophorus niveus*	*Cuphophyllus virgineus*	Sph	Toohey, (1983)	1983
Ascomycota	*Helvella*	*Helvella connivens*	*Helvella connivens*	Sph	Toohey, (1983)	1983
Ascomycota	*Helvella*	*Helvella crispa*	*Helvella crispa*	Sph	Toohey, (1983)	1983
Basidiomycota	*Hygrophorus*	*Hygrophorus russula*	*Hygrophorus erubescens*	Sym	Toohey, (1983)	1983
Basidiomycota	*Inocybe*	*Inocybe lacera*	*Inocybe lacera*	Sym	Toohey, (1983)	1983
Basidiomycota	*Lactarius*	*Lactarius resimus*	*Lactarius resimus*	Sym	Toohey, (1983)	1983
Basidiomycota	*Lepista*	*Tricholoma irinum*	*Lepista irina*	sph	Toohey, (1983)	1983
Basidiomycota	*Lepista*	*Clitocybe subconnexa*	*Lepista subconnexa*	Sph	Toohey, (1983)	1983
Basidiomycota	*Leucopaxillus*	*Leucopaxillus giganteus*	*Leucopaxillus giganteus*	Sph	Toohey, (1983)	1983
Basidiomycota	*Lycoperdon*	*Lycoperdon perlatum*	*Lycoperdon perlatum*	sph	Toohey, (1983)	1983
Basidiomycota	*Lyophyllum*	*Lyophyllum immundum*	*Lyophyllum immundum*	Sph	Toohey, (1983)	1983
Basidiomycota	*Marasmius*	*Marasmius oreades*	*Marasmius oreades*	sph	Toohey, (1983)	1983
Basidiomycota	*Paxillus*	*Paxillus involutus*	*Paxillus involutus*	Sph	Toohey, (1983)	1983
Basidiomycota	*Russula*	*Russula aeruginea*	*Russula aeruginea*	Sym	Toohey, (1983)	1983
Basidiomycota	*Russula*	*Russula aurantiolutea*	*Russula aurantiolutea*	Sym	Toohey, (1983)	1983
Basidiomycota	*Russula*	*Russula decolorans*	*Russula decolorans*	Sym	Toohey, (1983)	1983
Basidiomycota	*Russula*	*Russula densifolia*	*Russula densifolia*	Sym	Toohey, (1983)	1983
Basidiomycota	*Suillus*	*Suillus granulatus*	*Suillus granulatus*	Sym	Toohey, (1983)	1983
Basidiomycota	*Tricholoma*	*Tricholoma terreum*	*Tricholoma terreum*	Sym	Toohey, (1983)	1983
Basidiomycota	*Floccularia*	*Floccularia luteovirens*	*Floccularia luteovirens*	Sym	Xing, (2017)	2017

Basidiomycota	*Agaricus*	*Agaricus campestris*	*Agaricus campestris*	Sph	Xu, (2011)	2011
Basidiomycota	*Agaricus*	*Agaricus gennadii*	*Agaricus gennadii*	Sph	Yang, (2018a)	2018
Basidiomycota	*Agaricus*	*Agaricus gennadii*	*Agaricus gennadii*	Sph	Yang, (2018b)	2018
Basidiomycota	*Agaricus*	*Agaricus gennadii*	*Agaricus gennadii*	Sph	Yang, (2018c)	2018
Basidiomycota	*Agaricus*	*Agaricus campestris*	*Agaricus campestris*	Sph	Yang, (2019)	2019
Basidiomycota	*Agaricus*	*Agaricus gennadii*	*Agaricus gennadii*	Sph	Yang, (2019)	2019
Basidiomycota	*Marasmius*	*Marasmius oreades*	*Marasmius oreades*	Sph	York, (2000)	2000
Basidiomycota	*Leucocalocybe*	*Tricholoma monogolicum*	*Leucocalocybe mongolica*	Sph	Zhao, (2003)	2003
Basidiomycota	*Tricholoma*	*Tricholoma matzutake*	*Tricholoma nauseosmus*	Sym	Zhou, (2021)	2021
Basidiomycota	*Amanita*	*Amanita vittadini*	*Amanita vittadini*	Sph	Zotti 2024	2024
Basidiomycota	*Marasmius*	*Marasmius oreades*	*Marasmius oreades*	Sph	Zotti 2024	2024
Basidiomycota	*Spodocybe*	*Spodocybe collina*	*Spodocybe collina*	Sph	Zotti 2024	2024
Basidiomycota	*Agaricus*	*Agaricus crocodilinus*	*Agaricus crocodilinus*	Sph	Zotti in preparation	2025
Basidiomycota	*Amanita*	*Amanita phalloides*	*Amanita phalloides*	Sym	Zotti in preparation	2025
Basidiomycota	*Tricholosporu m*	*Tricholosporum goniospermum*	*Tricholosporum goniospermum*	Sph	Zotti in preparation	2025
Basidiomycota	*Agaricus*	*Agaricus arvensis*	*Agaricus arvensis*	Sph	Zotti, (2020)	2020
Basidiomycota	*Calocybe*	*Calocybe gambosa*	*Calocybe gambosa*	Sph	Zotti, (2021)	2021

List of records on FFR-forming fungi and their global distribution

Taxon	**Ecology**	**reference**	**Nation**	**Long**	**Lat**
Marasmius oreades	Sph	Abesha & Høiland(2003)	*Norway*	6,4510	58,0458
Calocybe gambosa	Sph	Ainsworth & Bysby, (1950)	*United Kingdom*	-	-
Marasmius oreades	Sph	Albrecht et al. (1951)	*United States*	-92,1912	38,5633
Agaricus arvensis	Sph	Atkinson (1900)*	United States	-	-
Marasmius oreades	Sph	Ayer et al(1989)	*Canada*	-113,3233	53,2927
Agaricus arvensis	Sph	Ballion (1906)*	*France*	-	-
Hydnum repandum	Ecm	Ballion (1906)*	*France*	-	-
Lycoperdon perlatum	Sph	Ballion (1906)*	*France*	-	-
Marasmius oreades	Sph	Ballion (1906)*	*France*	-	-
Morchella esculenta	Sph	Ballion (1906)*	*France*	-	-
Morchella semilibera	Sph	Ballion (1906)*	*France*	-	-
Suillus bovinus	Ecm	Ballion (1906)*	*France*	-	-
Leucopaxillus giganteus	Sph	Bayliss (1911)*	*United Kingdom*	-	-
Marasmius oreades	Sph	Bayliss (1911)*	*United Kingdom*	-	-
Calocybe gambosa	Sph	Bayliss-Elliot (1926)	*United Kingdom*	-1,5106	52,2034
Lepista nuda	Sph	Bayliss-Elliot (1926)	*United Kingdom*	-1,5106	52,2034
Lepista nuda	Sph	Bayliss-Elliot (1926)	*United Kingdom*	-1,5106	52,2034
Lepista panaeola	Sph	Bayliss-Elliot (1926)	*United Kingdom*	-1,5106	52,2034
Lycoperdon perlatum	Sph	Bayliss-Elliot (1926)	*United Kingdom*	-1,5106	52,2034
Marasmius oreades	Sph	Bayliss-Elliot (1926)	*United Kingdom*	-1,5106	52,2034
Marasmius oreades	Sph	Blenis et al. (1997)	*Canada*	-113,2950	53,3124

Marasmius oreades	Sph	Blenis et al. (2004)	*Canada*	-113,2950	53,3124
Marasmius oreades	Sph	Blenis et al. (2004)	*Canada*	-113,2950	53,3124
Agaricus campestris	Sph	Bonanomi et al. (2012)	*Italy*	12,4838	43,2681
Agaricus arvensis	Sph	Buckman (1870)*	United States	-	-
Lepista nuda	Sph	Buckman (1870)*	United States	-	-
Marasmius oreades	Sph	Buckman (1870)*	United States	-	-
Marasmius oreades	Sph	Burnett & Evans, (1966)	*United Kingdom*	-1,3623	55,2935
Marasmius oreades	Sph	Caspar & Spitteler (2015)	*Germany*	8,5054	53,6734
Floccularia luteovirens	Sph	Chen, (2000)	*China*	10,0206	37,2516
Lepista nuda	Sph	Choi et al. (2022)	Japan	-	-
Lepista sordida	Sph	Choi, (2010a)	*Japan*	138,2560	34,5749
Lepista sordida	Sph	Choi, (2010b)	*Japan*	138,2560	34,5749
Vascellum curtisii	Sph	Cocker & Couch (1928)	United States	-	-
Marasmius oreades	Sph	Cosby, (1959)	*United States*	-104,4863	47,5512
Hydnellum suaveolens	Ecm	Coulter et al. (1911)*	United States	-	-
Marasmius oreades	Sph	Coville (1898)*	United States	-	-
Leucocalocybe mongolica	Sph	Dhiao, (2004)	*China*	100,1400	34,3900
Marasmius oreades	Sph	Dickinson, (1979)	*United Kingdom*	0,2693	52,4731
Clitocybe nebularis	Sph	Dowson et al. (1989)	*United Kingdom*	-2,1837	51,1950
Agaricus xanthodermus	Sph	Du et al. (2024)	*China*	101,1900	37,3600
Leucocalocybe mongolica	Sph	Duan et al (2021)	*China*	116,2517	43,5560
Leucocalocybe mongolica	Sph	Duan et al. (2022)a,b	*China*	119,5400	49,7200
Leucocalocybe mongolica	Sph	Duan, (2021a)	*China*	119,2943	49,9165
Leucocalocybe mongolica	Sph	Duan, (2021b)	*China*	119,2943	49,9165
Agaricus arvensis	Sph	Edwards (1984)	*United Kingdom*	-1,3623	50,4926
Agaricus arvensis	Sph	Edwards (1988)	*United Kingdom*	-1,3623	50,4926
Lycoperdon sp.	Sph	Elliot, (2002)	*United States*	-80,3112	26,6362
Agaricus lilaceps	Sph	Espeland, (2013)	*Canada*	-104,4248	47,4233
Gymnopus dryophilus	Sph	Falandysz, (2013)	*China*	102,1035	29,1531
Gymnopus erythropus	Sph	Falandysz, (2013)	*China*	102,1035	29,1531
Marasmius oreades	Sph	Fidanza, (2007)	*United States*	-75,5537	40,2083
Marasmius oreades	Sph	Filer, (1965a)	*United States*	-122,8130	47,6173
Marasmius oreades	Sph	Filer, (1965b)	*United States*	-122,8130	47,6173
Marasmius oreades	Sph	Fisher, (1976)	*Canada*	-79,2255	43,3960
Marasmius oreades	Sph	Fox, (2006)	*United Kingdom*	-0,5555	51,2625
Mycena galopus	Sph	Frankland, (1998)	*United Kingdom*	-	-
Marasmius oreades	Sph	Gramss, (2005)	*Germany*	11,3734	50,5448
Infundibulicybe geotropa	Sph	Gregory (1982)*	*France*	6,4613	47,3913
Calocybe gambosa	Sph	Guminska, (1976)	*Poland*	20,2628	49,2413
Clitocybe rivulosa	Sph	Guminska, (1976)	*Poland*	20,2628	49,2413
Cystoderma amianthinum	Sph	Guminska, (1976)	*Poland*	20,2628	49,2413
Lactarius semisanguifluus	Ecm	Guminska, (1976)	*Poland*	20,2628	49,2413
Lepista nuda	Sph	Guminska, (1976)	*Poland*	20,2628	49,2413
Macrolepiota mastoidea	Sph	Guminska, (1976)	*Poland*	20,2628	49,2413

Macrolepiota procera	Sph	Guminska, (1976)	*Poland*	20,2628	49,2413
Marasmius oreades	Sph	Guminska, (1976)	*Poland*	20,2628	49,2413
Pseudoclitocybe expallens	Sph	Guminska, (1976)	*Poland*	20,2628	49,2413
Agaricus arvensis	Sph	Halinsky & Peterson, (1970)	*United States*	-74,2766	40,2910
Agaricus campestris	Sph	Halinsky & Peterson, (1970)	*United States*	-74,2766	40,2910
Calvatia cyathiformis	Sph	Halinsky & Peterson, (1970)	*United States*	-74,2766	40,2910
Clitocybe caespitosa	Sph	Halinsky & Peterson, (1970)	*United States*	-74,2766	40,2910
Clitocybe rivulosa	Sph	Halinsky & Peterson, (1970)	*United States*	-74,2766	40,2910
Leucoagaricus leucolithes	Sph	Halinsky & Peterson, (1970)	*United States*	-74,2766	40,2910
Leucopaxillus giganteus	Sph	Halinsky & Peterson, (1970)	*United States*	-74,2766	40,2910
Marasmius oreades	Sph	Halinsky & Peterson, (1970)	*United States*	-74,2766	40,2910
Panaeolina foenisecii	Sph	Halinsky & Peterson, (1970)	*United States*	-74,2766	40,2910
Panaeolus papilionaceus	Sph	Halinsky & Peterson, (1970)	*United States*	-74,2766	40,2910
Marasmius oreades	Sph	Hardwick & Heard, (1978)	*United Kingdom*	-0,2546	50,5552
Albatrellopsis confluens	Ecm	Hawksworth (1962)	*United States*	-105,3320	40,4060
Clitocybe nebularis	Sph	Hears et al. (2013)	*Northern Ireland*	-5,5647	54,3332
Marasmius oreades	Sph	Hiltunen et al. (2019)	*Sweden*	14,3922	58,1821
Maramius oreades	Sph	Hiltunen et al. (2021)	*Sweden*	17,3419	59,5127
Lepista nuda	Sph	Hjelm, (1994)	*Sweden*	15,5818	57,4544
Leucoagaricus leucothites	Sph	Howards et al. (1951)	United States	-	-
Macrolepiota procera	Sph	Howards et al. (1951)	United States	-	-
Marasmius oreades	Sph	Ingold, (1974)	*United Kingdom*	0,1929	51,1722
Floccularia luteovirens	Sph	Jinyang, (2005)	*China*	-	-
Agaricus campestris	Sph	Jorden (1862)*	United States	-	-
Leucopaxillus giganteus	Sph	Jorden (1862)*	United States	-	-
Tricholoma matsutake	Ecm	Kataoka et al. (2012)	*Japan*	135,3058	35,8771
Amanita muscaria	Ecm	Kauffman (1918)	United States	-	-
Tricholoma matsutake	Ecm	Kim, (2013)	*South Korea*	128,3790	35,4526
Tricholoma matsutake	Ecm	Kim, (2014)	*South Korea*	128,3790	35,4526
Tricholoma matsutake	Ecm	Koo et al. (2009)	*South Korea*	127,1818	36,5139
Clitocybe nebularis	Sph	Kosanic, (2020)	*Serbia*	20,5621	44,0308
Infundibulicybe geotropa	Sph	Kosanic, (2020)	*Serbia*	20,5621	44,0308
Pheolepiota aurea	Sph	Laessoe & Petersen 2019	Denmark	-	-
Hygrophorus agathosmus	Sph	Larsson, (2018)	*Sweden*	11,5857	57,4152
Marasmius oreades	Sph	Lawes et al. (1883)*	*United Kingdom*	-0,2124	51,4832
Calocybe gambosa	Sph	Lawes et al. (1883)*	*United Kingdom*	-0,2124	51,4832
Marasmius oreades	Sph	Lebeau, 1961	*Canada*	-112,4550	49,4156
Marasmius oreades	Sph	Lebeau, 1963	*Canada*	-112,4550	49,4156
Marasmius oreades	Sph	Lebeau, 1963b	*Canada*	-112,4550	49,4156
Agaricus arvensis	Sph	Lees (1869)*		-	-
Cantharellus cibarius	Ecm	Lees (1869)*		-	-
Collybia sp.	Sph	Lees (1869)*		-	-
Collybiopsis confluens	Sph	Lees (1869)*		-	-
Collybiopsis peronata	Sph	Lees (1869)*		-	-
Cuphophyllus virgineus	Sph	Lees (1869)*		-	-
Hebeloma crustuliniforme	Ecm	Lees (1869)*		-	-

Hydnum repandum	Ecm	Lees (1869)*		-	-
Infundibulicybe geotropa	Sph	Lees (1869)*		-	-
Infundibulicybe gibba	Sph	Lees (1869)*		-	-
Lactifluus piperatus	Ecm	Lees (1869)*		-	-
Lepista nuda	Sph	Lees (1869)*		-	-
Marasmius oreades	Sph	Lees (1869)*		-	-
Melanoleuca grammopodia	Sph	Lees (1869)*		-	-
-	Sph	Li et al. (2022)	China	115,4000	41,4600
Tricholoma matsutake	Ecm	Li, (2016)	*China*	102,1037	28,3311
Tricholoma matsutake	Ecm	Lian et al. (2006)	*Japan*	141,1400	39,5600
Agaricus bisporus	Sph	Liu et al. (2021)	*China*	-99,2644	38,3378
Agaricus campestris	Sph	Liu et al. (2023)	China	120,2200	50,4000
Lepista sordida	Sph	Liu et al. (2023)	China	120,2200	50,4000
Leucocalocybe mongolica	Sph	Liu et al. (2023)	China	120,2200	50,4000
Amanita muscaria	Ecm	Ludgwig (1906)*	Germany	-	-
Amanita phalloides	Ecm	Ludgwig (1906)*	Germany	-	-
Cantharellus cinereus	Ecm	Ludgwig (1906)*	Germany	-	-
Cortinarius armillatus	Ecm	Ludgwig (1906)*	Germany	-	-
Cortinarius traganus	Ecm	Ludgwig (1906)*	Germany	-	-
Hydnum compactum	Ecm	Ludgwig (1906)*	Germany	-	-
Hygrophoropsis aurantiaca	Sph	Ludgwig (1906)*	Germany	-	-
Lactarius insulsus	Ecm	Ludgwig (1906)*	Germany	-	-
Lactarius torminosus	Ecm	Ludgwig (1906)*	Germany	-	-
Marasmius oreades	Sph	Ludgwig (1906)*	Germany	-	-
Paxillus involutus	Sph	Ludgwig (1906)*	Germany	-	-
Suillus cavipes	Ecm	Ludgwig (1906)*	Germany	-	-
Suillus elegnas	Ecm	Ludgwig (1906)*	Germany	-	-
Suillus variegatus	Ecm	Ludgwig (1906)*	Germany	-	-
Marasmius oreades	Sph	Mallet & Harrison (1988)	*Canada*	-115,3135	52,4185
Agaricus campestris	Sph	Massart (1910)*	*France*	-	-
Marasmius oreades	Sph	Massart (1910)*	*France*	-	-
Tricholoma columbetta	Ecm	Massart (1910)*	*France*	-	-
Marasmius oreades	Sph	Mathur, (1970)	*Canada*	-75,4348	45,2259
Lycoperdon cyclicum	Sph	McAlpine (1898)*	*United States*	-	-
Marasmius oreades	Sph	Miles & Mathur (1971)	*Canada*	-75,4348	45,2259
Agaricus argenteus	Sph	Miller and Gongloff, (2021)	*United States*	-105,4346	41,1754
Agaricus lilaceps	Sph	Miller and Gongloff, (2021)	*United States*	-105,4346	41,1754
Bovista plumbea	Sph	Miller and Gongloff, (2021)	*United States*	-105,4346	41,1754
Calvatia chilensis	Sph	Miller and Gongloff, (2021)	*United States*	-105,4346	41,1754
Calvatia cyathiformis	Sph	Miller and Gongloff, (2021)	*United States*	-105,4346	41,1754
Disciseda candida	Sph	Miller and Gongloff, (2021)	*United States*	-105,4346	41,1754
Geastrum sp.	Sph	Miller and Gongloff, (2021)	*United States*	-105,4346	41,1754
Lepiota erminea	Sph	Miller and Gongloff, (2021)	*United States*	-105,4346	41,1754
Leucocalocybe mongolica	Sph	Miller and Gongloff, (2021)	*United States*	-105,4346	41,1754
Lycoperdon dermoxanthum	Sph	Miller and Gongloff, (2021)	*United States*	-105,4346	41,1754

Marasmius oreades	Sph	Miller and Gongloff, (2021)	*United States*	-105,4346	41,1754
Tulostoma calcareum	Sph	Miller and Gongloff, (2021)	*United States*	-105,4346	41,1754
Agaricus argenteus	Sph	Miller and Gongloff, (2023)	*United States*	-105,4346	41,1754
Agaricus lilaceps	Sph	Miller and Gongloff, (2023)	*United States*	-105,4346	41,1754
Bovista plumbea	Sph	Miller and Gongloff, (2023)	*United States*	-105,4346	41,1754
Calvatia spp.	Sph	Miller and Gongloff, (2023)	*United States*	-105,4346	41,1754
Disciseda candida	Sph	Miller and Gongloff, (2023)	*United States*	-105,4346	41,1754
Geastrum sp.	Sph	Miller and Gongloff, (2023)	*United States*	-105,4346	41,1754
Marasmius oreades	Sph	Miller and Gongloff, (2023)	*United States*	-105,4346	41,1754
Agaricus arvensis	Sph	Miller et al. (2012)	*United States*	-78,4116	35,4347
Agaricus campestris	Sph	Miller et al. (2012)	*United States*	-78,4116	35,4347
Arachnion album	Sph	Miller et al. (2012)	*United States*	-78,4116	35,4347
Coprinopsis kubickae	Sph	Miller et al. (2012)	*United States*	-78,4116	35,4347
Coprinus comatus	Sph	Miller et al. (2012)	*United States*	-78,4116	35,4347
Coprinus sp	Sph	Miller et al. (2012)	*United States*	-78,4116	35,4347
Cyclocybe cilindracea	Sph	Miller et al. (2012)	*United States*	-78,4116	35,4347
Lepista nuda	Sph	Miller et al. (2012)	*United States*	-78,4116	35,4347
Lepista sordida	Sph	Miller et al. (2012)	*United States*	-78,4116	35,4347
Lycoperdon curtisii	Sph	Miller et al. (2012)	*United States*	-78,4116	35,4347
Lycoperdon dermoxanthum	Sph	Miller et al. (2012)	*United States*	-78,4116	35,4347
Lycoperdon dermoxanthum	Sph	Miller et al. (2012)	*United States*	-78,4116	35,4347
Lycoperdon marginatum	Sph	Miller et al. (2012)	*United States*	-78,4116	35,4347
Marasmius graminum	Sph	Miller et al. (2012)	*United States*	-78,4116	35,4347
Marasmius oreades	Sph	Miller et al. (2012)	*United States*	-78,4116	35,4347
Marasmius siccus	Sph	Miller et al. (2012)	*United States*	-78,4116	35,4347
Marasmius sp.	Sph	Miller et al. (2012)	*United States*	-78,4116	35,4347
Parasola pliicatilis	Sph	Miller et al. (2012)	*United States*	-78,4116	35,4347
Collybia pinastris	Sph	Miyamoto, (2004)	*Japan*	142,1443	43,4054
Marasmius oreades	Sph	Molliard (1910)*	*France*	-	-
Marasmius oreades	Sph	Molliard 1910	*France*	-2,1449	48,3843
Clavaria sp.	Sph	Munch (1914)*	*Germany*	-	-
Infundibulicybe geotropa	Sph	Munch (1914)*	*Germany*	-	-
Lepista nuda	Sph	Munch (1914)*	*Germany*	-	-
Tricholoma matsutake	Ecm	Narimatsu, (2015)	*Japan*	141,1400	39,5600
Marasmius oreades	Sph	Northstat, (1973)	*United States*	-105,1316	39,4520
Lepiota sp.	Sph	Odamtten et al. (2022)	*Ghana*	0,4288	5,4851
Macrolepiota procera	Sph	Odamtten et al. (2022)	Ghana	0,4288	5,4851
Tricholoma matsutake	Ecm	Oh et al. (2016)	*South Korea*	129,1348	35,4846
Tricholoma matsutake	Ecm	Oh et al. (2018)	*South Korea*	127,5851	37,4135
Tricholoma matsutake	Ecm	Ohara & Hamada (1967)	*Japan*	135,4497	35,6179
Agaricus sp.	Sph	Olivier (1891)*	*France*	-	-
Calocybe gambosa	Sph	Ramsbolton (1953)	*United Kingdom*	-	-
Infundibulicybe geotropa	Sph	Ramsbolton (1953)	*United Kingdom*	-	-
Infundibulicybe geotropa	Sph	Rea (1922)	*United Kingdom*	-	-
Coprinopsis kubickae	Sph	Redhead & Smith (1981)	*Canada*	-106,2731	52,5624
Marasmius oreades	Sph	Reed (1910)*	*United States*	-80,2524	37,1342

Agaricus campestris	Sph	Ritsema Bos (1901)*	*Germany*	-	-
Leucopaxillus giganteus	Sph	Ritsema Bos (1901)*	*Germany*	-	-
Marasmius oreades	Sph	Ritsema Bos (1901)*	*Germany*	-	-
-	Sph	Rodriguez et al. (2022)	Spain	1,3940	42,0556
Agaricus arvensis	Sph	Rogers & McAllister (1969)	*South Africa*	28,5566	-28,4239
Agaricus campestris	Sph	Shantz and Piezemel, (1917)	*United States*	-103,1419	40,9135
Agaricus praerimosus	Sph	Shantz and Piezemel, (1917)	*United States*	-103,1419	40,9135
Calvatia cyathiformis	Sph	Shantz and Piezemel, (1917)	*United States*	-103,1419	40,9135
Calvatia cyathiformis	Sph	Shantz and Piezemel, (1917)	*United States*	-103,1419	40,9135
Calvatia polygonia	Sph	Shantz and Piezemel, (1917)	*United States*	-103,1419	40,9135
Chlorophyllum morganii	Sph	Shantz and Piezemel, (1917)	*United States*	-103,1419	40,9135
Disciseda subterranea	Sph	Shantz and Piezemel, (1917)	*United States*	-103,1419	40,9135
Lycoperdon wrightii	Sph	Shantz and Piezemel, (1917)	*United States*	-103,1419	40,9135
Melanoleuca malaleuca	Sph	Shantz and Piezemel, (1917)	*United States*	-103,1419	40,9135
Clitopilus prunulus	Sph	Shantz and Piezemel, (1917)*	-	-	-
Pluteus cervinus	Sph	Shantz and Piezemel, (1917)*	-	-	-
Tricholoma equestre	Ecm	Shantz and Piezemel, (1917)*	-	-	-
Lepista luscina	Sph	Siyu et al. (2023)	Mongolia	99,4590	50,4361
Agaricus arvensis	Sph	Smith (1957)	United States	-	-
Agaricus campestris	Sph	Smith (1957)	United States	-	-
Gliophorus psittacinus	Sph	Smith (1957)	United States	-	-
Lepista nuda	Sph	Smith (1957)	United States	-	-
Marasmius oreades	Sph	Smith (1957)	United States	-	-
Rhodocollybia butirracea	Sph	Smith (1957)	United States	-	-
Scleroderma verrucosum	Ecm	Smith (1957)	United States	-	-
Calvatia cyathiformis	Sph	Sprague (1946)	United States	-	-
Clitocybe nebularis	Sph	Stahl (1900)*	*Germany*	-	-
Lycoperdon dermoxanthum	Sph	Terashima et al. (2004)	*Japan*	140,6235	35,3627
Vascellum curtisii	Sph	Terashima et al. (2004)	*Japan*	141,6235	35,3627
Lepista sordida	Sph	Terashima et al. (2007)	*Japan*	142,6235	35,3627
Hydnellum suaveolens	Ecm	Thomas (1905)*	*Germany*	-	-
Agaricus campestris	Sph	Toohey, (1983)	*Canada*	-76,1958	44,3323
Agaricus xanthodermus	Sph	Toohey, (1983)	*Canada*	-76,1958	44,3323
Amanita flavorubescens	Ecm	Toohey, (1983)	*Canada*	-76,1958	44,3323
Amanita muscaria	Ecm	Toohey, (1983)	*Canada*	-76,1958	44,3323
Calvatia cyathiformis	Sph	Toohey, (1983)	*Canada*	-76,1958	44,3323
Cantharellus cibarius	Ecm	Toohey, (1983)	*Canada*	-76,1958	44,3323
Clavaria fragilis	Sph	Toohey, (1983)	*Canada*	-76,1958	44,3323
Clavulina cinerea	Sph	Toohey, (1983)	*Canada*	-76,1958	44,3323
Clitocybe rivulosa	Sph	Toohey, (1983)	*Canada*	-76,1958	44,3323
Cortinarius sp	Ecm	Toohey, (1983)	*Canada*	-76,1958	44,3323
Cortinarius sp 1	Sph	Toohey, (1983)	*Canada*	-76,1958	44,3323
Cuphophyllus virgineus	Sph	Toohey, (1983)	*Canada*	-76,1958	44,3323

Helvella connivens	Sph	Toohey, (1983)	*Canada*	-76,1958	44,3323
Helvella crispa	Sph	Toohey, (1983)	*Canada*	-76,1958	44,3323
Hygrophorus erubescens	Ecm	Toohey, (1983)	*Canada*	-76,1958	44,3323
Inocybe lacera	Ecm	Toohey, (1983)	*Canada*	-76,1958	44,3323
Lactarius resimus	Ecm	Toohey, (1983)	*Canada*	-76,1958	44,3323
Lepista irina	Sph	Toohey, (1983)	*Canada*	-76,1958	44,3323
Lepista subconnexa	Sph	Toohey, (1983)	*Canada*	-76,1958	44,3323
Leucopaxillus giganteus	Sph	Toohey, (1983)	*Canada*	-76,1958	44,3323
Lycoperdon perlatum	Sph	Toohey, (1983)	*Canada*	-76,1958	44,3323
Lyophyllum immundum	Sph	Toohey, (1983)	*Canada*	-76,1958	44,3323
Marasmius oreades	Sph	Toohey, (1983)	*Canada*	-76,1958	44,3323
Paxillus involutus	Sph	Toohey, (1983)	*Canada*	-76,1958	44,3323
Russula aeruginea	Ecm	Toohey, (1983)	*Canada*	-76,1958	44,3323
Russula aurantiolutea	Ecm	Toohey, (1983)	*Canada*	-76,1958	44,3323
Russula decolorans	Ecm	Toohey, (1983)	*Canada*	-76,1958	44,3323
Russula densifolia	Ecm	Toohey, (1983)	*Canada*	-76,1958	44,3323
Suillus granulatus	Ecm	Toohey, (1983)	*Canada*	-76,1958	44,3323
Tricholoma terreum	Ecm	Toohey, (1983)	*Canada*	-76,1958	44,3323
Tuber sp.	Ecm	Toulasne & Toulasne (1851)*	*France*	-	-
Agaricus campestris	Sph	Van Tieghmen (1884)*	*France*	-	-
Leucopaxillus giganteus	Sph	Van Tieghmen (1884)*	*France*	-	-
Marasmius oreades	Sph	Van Tieghmen (1884)*	*France*	-	-
Leucocalocybe mongolica	Sph	Wang et al. (2022)a,b	China	84,9215	42,5642
Calocybe gambosa	Sph	Way (1847)*	*United Kingdom*	-	-
Chlorophyllum morganii	Sph	Williams (1897)*	*United Kingdom*	-	-
Inocybe sp.	Ecm	Williams (1897)*	*United Kingdom*	-	-
Marasmius oreades	Sph	Williams (1897)*	*United Kingdom*	-	-
Tricholoma sp.	Ecm	Willimas (1901)*	*United Kingdom*	-	-
Tricholoma terreum	Ecm	Winslow (1954)	United States	-	-
Marasmius oreades	Sph	Withering (1796)*	*United Kingdom*	-	-
Agaricus campestris	Sph	Wollaston et al. (1807)*	*United Kingdom*	-3,9430	55,5639
Bovista plumbea	Sph	Wollaston et al. (1807)*	*United Kingdom*	-3,9430	55,5639
Macrolepiota procera	Sph	Wollaston et al. (1807)*	*United Kingdom*	-3,9430	55,5639
Marasmius oreades	Sph	Wollaston et al. (1807)*	*United Kingdom*	-3,9430	55,5639
Tricholoma terreum	Ecm	Wollaston et al. (1807)*	*United Kingdom*	-3,9430	55,5639
Floccularia luteovirens	Sph	Xing et al. (2022)	China	99,2926	38,3130
Floccularia luteovirens	Ecm	Xing, (2017)	*China*	99,2926	38,3130
Agaricus campestris	Sph	Xu, (2011)	*China*	101,1914	37,3660
Agaricus gennadii	Sph	Yang, (2018a)	*China*	115,4000	41,4400
Agaricus gennadii	Sph	Yang, (2018b)	*China*	115,4000	41,4400
Agaricus gennadii	Sph	Yang, (2018c)	*China*	115,4000	41,4400
Agaricus campestris	Sph	Yang, (2019)	*China*	115,4000	41,4400
Agaricus gennadii	Sph	Yang, (2019)	*China*	115,4000	41,4400
Marasmius oreades	Sph	York & Canaway (2000)	*United Kingdom*	-0,2942	54,1125
Leucocalocybe mongolica	Sph	Zhao, (2003)	*China*	99,1800	25,1600
Tricholoma matsutake	Ecm	Zhou, (2021)	*China*	101,0000	25,1000

Amanita vittadini	Sph	Zotti 2024	*Italy*	14,1936	41,4376
Marasmius oreades	Sph	Zotti 2024	*Italy*	14,1936	42,4376
Spodocybe collina	Sph	Zotti 2024	*Italy*	14,1936	43,4376
Agaricus crocodilinus	Sph	Zotti in preparation	*Italy*	13,5858	41,5031
Tricholosporum goniospermum	Sph	Zotti in preparation	*Italy*	13,5858	41,5031
Amanita phalloides	Ecm	Zotti in preparation	*Italy*	14,2042	40,4840
Agaricus arvensis	Sph	Zotti, (2020)	*Italy*	12,5130	43,1734
Calocybe gambosa	Sph	Zotti, (2021)	*Spain*	-5,3524	42,3460

List of records on FFR-forming fungi and relative depth in soil

species	depth	environment	reference
Agaricus campestris	24,5	Turf grasses	Terrashima, 2004
Tricholoma matzutake	20	Turf grasses	Terrashima, 2004
Tricholoma matzutake	20	ECM woodland	Ohara, 1967
Marasmius oreades	20	ECM woodland	Kataoka, 2011
Tricholoma matzutake	15	ECM woodland	Yamananka,2020
Marasmius oreades	14,5	Pastures	Cosby, 1959
Marasmius oreades	12,7	Pastures	Hardwick, 1978
Agaricus arvensis	10	Managed grassland	Edwards, 1984
Agaricus arvensis	8	Managed grassland	Edwards, 1988
Bovista dermoxanta	4	Managed grassland	Bonanomi, 2012
Vascellum curtisii	2	Turf grasses	Terrashima, 2007
Lepista sordida	2	Pastures	Nordstat, 1973

List of records on FFR-forming fungi and relative growth rate

species	classification	cm per years	environment	Ecology	Reference
Clitocybe nebularis	*Clitocybe*	90	Woodland	Sph	Dowson, 1998
Clitocybe dealbata	*Clitocybe*	75	Grassland	Sph	Toohey, 1983
Clitocybe subconnexa	*Clitocybe*	60	Grassland	Sph	Toohey, 1983
Lepista irina	*Lepista*	60	Woodland	Sph	Toohey, 1983
Lepista sordida	*Lepista*	125	Turf grasses	Sph	Terrashima, 2007
Lepista sordida	*Lepista*	17,3	Pastures	Sph	Elliot, 1926
Agaricus argenteus	*Agaricus*	26	Grassland	Sph	Miller and Gongloff (2023)
Agaricus arvensis	*Agaricus*	60	Managed grassland	Sph	Edwards, 1984
Agaricus arvensis	*Agaricus*	60	Managed grassland	Sph	Edwards, 1984
Agaricus arvensis	*Agaricus*	50	Pastures	Sph	Ballion, 1906
Agaricus campestris	*Agaricus*	60	Managed grassland	Sph	Bonanomi, 2012
Agaricus campestris	*Agaricus*	45	Grassland	Sph	Toohey, 1983
Agaricus lilaceps	*Agaricus*	26	Grassland	Sph	Miller and Gongloff (2023)
Agaricus tabularis	*Agaricus*	12	Pastures	Sph	Shantz and piemeisel, 1917
Agaricus xanthodermus	*Agaricus*	24	Woodland	Sph	Toohey, 1983

Marasmius oreades	*Marasmius*	62	Pastures	Sph	Bayliss, 1911
Marasmius oreades	*Marasmius*	35	Golg courses	Sph	Smith, 1957
Marasmius oreades	*Marasmius*	35	Pastures	Sph	Hardwick, 1978
Marasmius oreades	*Marasmius*	34,29	Pastures	Sph	Cosby, 1959
Marasmius oreades	*Marasmius*	32,5	Garden	Sph	Ingold, 1974
Marasmius oreades	*Marasmius*	22	Grassland	Sph	Toohey, 1983
Marasmius oreades	*Marasmius*	15,5	Turf grasses	Sph	Wolf, 1971
Marasmius oreades	*Marasmius*	15	Pastures	Sph	Nordstat, 1973
Marasmius oreades	*Marasmius*	13,5	Pastures	Sph	Dickinson, 1979
Marasmius oreades	*Marasmius*	12	Pastures	Sph	Ballion, 1906
Marasmius oreades	*Marasmius*	26	Grassland	Sph	Miller and Gongloff (2023)
Clavaria cinerea	*SPH other*	7	Grassland	Sph	Toohey, 1983
Clavaria vermicularis	*SPH other*	30	Grassland	Sph	Toohey, 1983
Cuphophillus virgineus	*SPH other*	20	Grassland	Sph	Toohey, 1983
Disciseda candida	*SPH other*	36,4	Grassland	Sph	Miller and Gongloff (2023)
Geastrum sp.	*SPH other*	13,1	Grassland	Sph	Miller and Gongloff (2023)
Helvella connivens	*SPH other*	30	Woodland	Sph	Toohey, 1983
Helvella crispa	*SPH other*	32	Woodland	Sph	Toohey, 1983
Hydnum suaveolens	*SPH other*	23	Woodland	Sph	Thomas, 1896
Bovista plumbea	*SPH other*	27	Grassland	Sph	Miller and Gongloff (2023)
Lycoperdon perlatum	*SPH other*	35	Grassland	Sph	Toohey, 1983
Lyophyllum immundum	*SPH other*	10	Grassland	Sph	Toohey, 1983
Calvatia spp.	*Calvatia*	27	Grassland	Sph	Miller and Gongloff (2023)
Calvatia cyathiformis	*Calvatia*	24	Pastures	Sph	Shantz and piemeisel, 1917
Calvatia fragilis	*Calvatia*	20	Grassland	Sph	Toohey, 1983
Russula aeruginosa	*Russula*	32	Woodland	Ecm	Toohey, 1983
Russula decolorans	*Russula*	20	Woodland	Ecm	Toohey, 1983
Russula densifolia	*Russula*	20	Woodland	Ecm	Toohey, 1983
Paxillus involutus	*ECM other*	25	Woodland	Sph	Toohey, 1983
Suillus granulatus	*ECM other*	30	Woodland	Ecm	Toohey, 1983
Inocybe lacera	*ECM other*	20	Woodland	Ecm	Toohey, 1983
Cantharellus cibarius	*ECM other*	19	Woodland	Ecm	Toohey, 1983
Cortinarius sp.	*ECM other*	15	Woodland	Ecm	Toohey, 1983
Amanita flavorubescens	*ECM other*	40	Woodland	Ecm	Toohey, 1983
Amanita muscaria	*ECM other*	10	Woodland	Ecm	Toohey, 1983
Tricholoma nauseosmus	*Tricholoma*	17	ECM woodland	Ecm	Kawakami, 1994
Tricholoma nauseosmus	*Tricholoma*	17	ECM woodland	Ecm	Narimatsu, 2015
Tricholoma nauseosmus	*Tricholoma*	15,7	ECM woodland	Ecm	Lian 2006,
Tricholoma nauseosmus	*Tricholoma*	15	ECM woodland	Ecm	Kataoka, 2011
Tricholoma nauseosmus	*Tricholoma*	15	ECM woodland	Ecm	Ogawa, 1975
Tricholoma nauseosmus	*Tricholoma*	12,5	Woodland	Ecm	Kim 2013
Tricholoma nauseosmus	*Tricholoma*	12	Woodland	Ecm	Koo 2009
Tricholoma nauseosmus	*Tricholoma*	10	ECM woodland	Ecm	Ohara, 1967
Tricholoma nauseosmus	*Tricholoma*	10	ECM woodland	Ecm	Yamananka,2020
Tricholoma terreum	*Tricholoma*	60	Woodland	Ecm	Toohey, 1983
